# Engineering Ferroelectricity and Large Piezoelectricity in h-BN

**DOI:** 10.1021/acsami.3c07744

**Published:** 2023-08-31

**Authors:** Mohammad Noor-A-Alam, Michael Nolan

**Affiliations:** Tyndall National Institute, University College Cork, Lee Maltings, Dyke Parade, Cork T12 R5CP, Ireland

**Keywords:** ferroelectric, piezoelectric, hexagonal boron nitride, DFT, wurtzite nitrides, negative piezoelectric constants, electric auxetic piezoelectric

## Abstract

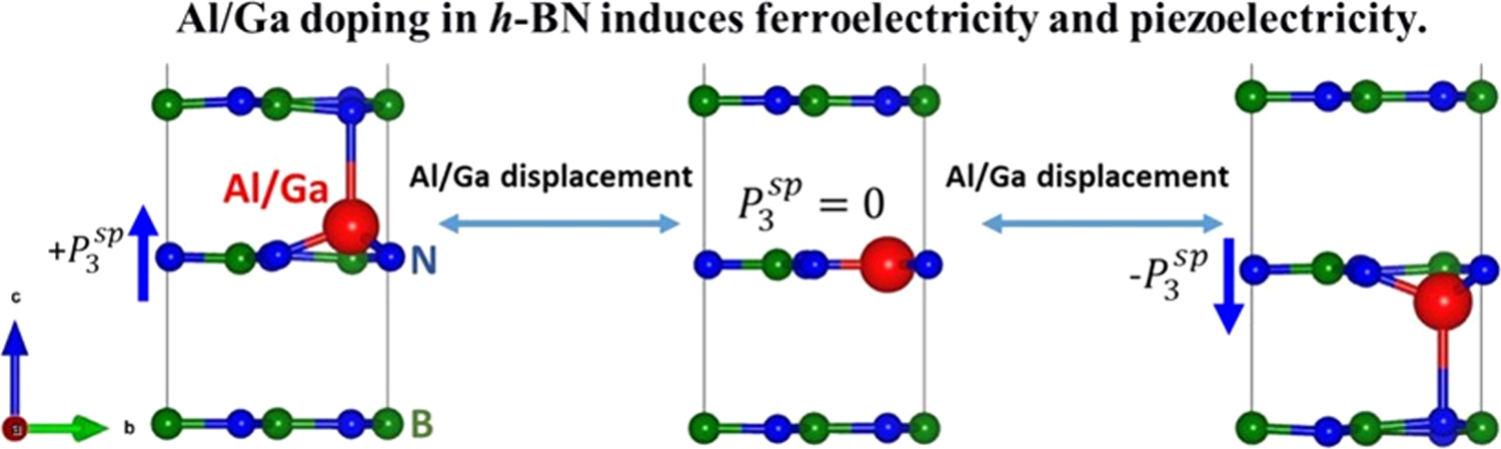

Hexagonal boron nitride (h-BN) is a well-known layered van der Waals (vdW) material that exhibits no spontaneous electric polarization due to its centrosymmetric structure. Extensive density functional theory (DFT) calculations are used to demonstrate that doping through the substitution of B by isovalent Al and Ga breaks the inversion symmetry and induces local dipole moments along the *c*-axis, which promotes a ferroelectric (FE) alignment over antiferroelectric. For doping concentrations below 25%, a “protruded layered” structure in which the dopant atoms protrude out of the planar h-BN layers is energetically more stable than the flat layered structure of pristine h-BN or a wurtzite structure similar to w-AlN. The computed polarization, between 7.227 and 21.117 μC/cm^2^, depending on dopant concentration and the switching barrier (16.684 and 45.838 meV/atom) for the FE polarization reversal are comparable to that of other well-known FEs. Interestingly, doping of h-BN also induces a large negative piezoelectric response in otherwise nonpiezoelectric h-BN. For example, we compute *d*_33_ of −24.214 pC/N for Ga_0.125_B_0.875_N, which is about 5 times larger than that of pure w-AlN (5 pC/N), although the computed *e*_33_ (−1.164 C/m^2^) is about 1.6 times lower than that of pure w-AlN (1.462 C/m^2^). Because of the layered structure, the rather small elastic constant *C*_33_ provides the origin of the large *d*_33_. Moreover, doping makes h-BN an electric auxetic piezoelectric. We also show that ferroelectricity in doped h-BN may persist down to its trilayer, which indicates high potential for applications in FE nonvolatile memories.

## Introduction

Ferroelectric (FE) memory devices including FE tunnel junctions (FTJs) and FE field effect transistors (FeFET) are identified as exciting platforms to realize brain-inspired and low-power neuromorphic computing.^1−3^ Developing new FEs that are compatible with the complementary metal oxide semiconductor (CMOS) process has been a long-lasting ambition, both technologically and scientifically. Unfortunately, the well-known perovskite oxide FEs such as PbZrTiO_3_ (PZT) are not CMOS-compatible and generally show a critical thickness limit below which ferroelectricity disappears due to the unscreened depolarization field.^4^ In this regard, the recent discovery of ferroelectricity in CMOS-compatible doped w-AlN is highly promising because of its high polarization and Curie temperature.^5−8^ w-AlN possesses large spontaneous polarization (130 μC/cm^2^) when hexagonal AlN is considered as paraelectric phase.^7,8^ However, because of the high coercive field, which is larger than its dielectric breakdown voltage, FE switching in pure w-AlN is impractical. Promisingly, substitution of Al by Sc or codoping of Li and V/Nb/Ta is an effective way to lower the polarization switching voltage.^5−8^ Recently, ferroelectricity with large polarization (125 μC/cm^2^) was demonstrated in boron-substituted w-AlN thin films,^9^ with boron concentrations in the range of 2–15%. However, a degradation in the ferroelectric response has been reported for B concentrations above 15%. Unlike AlN, BN has a centrosymmetric planar hexagonal crystal structure, and therefore h-BN is non-FE and nonpiezoelectric. In this paper, we pose the research question: can the substitution of boron by another trivalent element be used to engineer ferroelectricity and piezoelectricity in h-BN by breaking its centrosymmetry?

h-BN is a layered material with high thermal and chemical stability, widely used as dielectric substrate and protective coating layers in two-dimensional (2D) material-based devices,^3,10^ e.g., graphene/h-BN FET.^11^ Due to its van der Waals layered structure, h-BN can be synthesized as few-layer or even single-layer sheets. This makes it compatible with the scaling trend in the CMOS technology, where thinner layers are desired for miniaturization and improved device performance. Therefore, engineering ferroelectricity in h-BN nanofilms can integrate graphene-like high-mobility materials into FeFET, where the absence of dangling bonds at the interfaces protects their individual properties.^12^ Also for FTJs, FE h-BN nanofilms can be an ideal FE tunnel barrier material due to their uniform atomic thickness.^12^ Moreover, because of its compatibility with other 2D materials, piezoelectricity of FE h-BN can be ideal for piezotronic nanodevices—for example, strain-controlled transistors or strain-gated memory devices, where mechanical strain is used to modulate the electrical behavior and control device functionalities.^13^ Furthermore, it is noteworthy to mention that in recent developments, an approximately 18-layer-thick h-BN (roughly 6 nm) sheet has been successfully transferred onto the back-end-of-line interconnections of silicon microchips that contain CMOS transistors of the 180 nm node. This transfer was carried out specifically for memristive applications.^14^

Many modern devices such as resonators for radio frequency filtering, medical ultrasound, fuel injectors, sonar, and vibration-powered electronics rely on the piezoelectric property of materials that can convert mechanical to electrical energy, and vice versa. The piezoelectric coefficients *d*_*ij*_ and *e*_*ij*_ quantify the strength of the piezo-response. Usually, the longitudinal strain piezo-coefficient *d*_33_ of common piezoelectrics, e.g., PbTiO_3_, is positive as the material expands along the polar axis under an applied electric field in the same direction. However, recently negative piezo-response has been discovered in layered materials, where the material instead shrinks under an external electric field.^15−20^ This negative piezo-response may offer novel avenues for designing electromechanical devices. For example, an FE with a negative longitudinal stress piezo-coefficient, *e*_33_, can find high-pressure sensor applications because of its pressure-enhanced polarization. Being an intrinsically layered material, negative longitudinal piezoelectric constants (*e*_33_ and *d*_33_) can be engineered in h-BN, by breaking its inversion symmetry. For example, in recent experiments, layer sliding, in which one layer of h-BN slides against a neighboring layer in bilayer h-BN, has been employed to break the inversion symmetry and thus obtain an out-of-plane spontaneous polarization.^12^ Redistribution of the electron charge density due to different stacking during the sliding is the origin of the FE polarization in this case. Interestingly, a small negative longitudinal piezoelectric response (*d*_33_ = −0.97 pm/V) has been predicted for the FE stacking (AB or BA type stacking).^20^ However, engineering atomic level sliding in a controlled manner is not really practical for many applications such as FeFET. In addition, the piezoelectric response should be much larger, e.g., *d*_33_ for w-AlN is 5 pC/N,^21^ which is enhanced to approximately 25 pC/N at around 27% Sc doping.^22^

In this work, we show that ferroelectricity and a large piezoelectric response can be introduced into h-BN through substitutional doping of B by isovalent Al and Ga. This breaks the inversion symmetry as Al/Ga atoms protrude out of the flat plane of h-BN due to local sp^3^-like Al/Ga–N bonds, inducing a local electric dipole moment, which we find switchable with energy barriers in the range of 16.684–45.838 meV/atom, which yield facile ferroelectric switching. Surprisingly and of high relevance, we discover that these doped h-BN exhibits large negative *d*_33_ (−24.214 pC/N), which is significantly larger than in commercially used w-AlN piezoelectric (5 pC/N^21^). Moreover, ferroelectricity can persist down to trilayer thickness, thus eliminating the problem of reduced FE response as a result of the depolarization field found in other FE materials at this scale. We propose that FE-doped h-BN can find widespread applications in nonvolatile FE memories, particularly in piezoelectric applications such as energy harvesting or sensors.

## Computational Method

Our first-principles calculations are performed in the framework of spin-polarized density functional theory using projector augmented wave (PAW) potentials to describe the core electrons and the generalized gradient approximation (GGA) of Perdew, Burke, and Ernzernhof (PBE) for exchange and correlation as implemented in the Vienna Ab initio Simulation Package (VASP)^23−25^ based on a plane-wave basis set. A cutoff energy of 500 eV for the plane-wave expansion is used in all calculations, and all structures are fully relaxed until the Hellmann–Feynman forces on all of the atoms are less than 0.01 eV/Å. The lattice parameters and internal coordinates of the structures are fully relaxed to achieve the lowest energy configurations using the conjugate gradient algorithm. Geometry optimization is carried out employing the conjugated gradient technique, and the convergence for the total energy is set as 10^–6^ eV.

The 2 × 2 × 2 supercells with 32 atoms are generated, and 1 B is replaced by 1 Al or 1 Ga atom for *x* = 0.0625 composition, whereas 2 B atoms are randomly substituted by 2 Al or 2 Ga for *x* = 0.125 composition. The PBE-D3 method^26^ is employed to account for the van der Waals interaction. We also consider doping in 3 × 3 × 2 and 2 × 2 × 4 supercells, and results are shown in Supporting Information. The structural data of the relaxed supercells are available in the Supporting Information and also at https://github.com/MMD-group/VASP. With In doping, no band gap is observed; hence, we have not studied In-doped h-BN further as typically ferroelectric/piezoelectric materials have a nonzero band gap. Density of states (DOS) of Al/Ga-doped h-BN is shown in the Supporting Information. We find that Al/Ga doping reduces the wide band gap of pristine h-BN. Al_0.0625_B_0.9375_N, Al_0.1250_B_0.8750_N, Ga_0.0625_B_0.9375_N, and Ga_0.1250_B_0.8750_N have a band gap of 1.18 eV. 1.73, 1.31, and 1.74 eV, respectively. Our calculated band gap of pristine bulk h-BN (4.40 eV) is quite lower than the experimental value (5.96 eV).^27^ This is expected as it is well known that the GGA-PBE functional underestimates the band gap. Therefore, we expect a larger experimental band gap for the doped h-BN. The resulting band gaps of doped h-BN are larger than the band gaps (0.5–1 eV) of *ABC* hexagonal hyper ferroelectric^28^ or the band gap of 2D ferroelectric In_2_Se_3_ layer (0.78 eV by GGA-PBE)^29^ and are comparable to that of BaTiO_3_ (1.56 eV obtained by GGA-PBE).^30^ Here, it is worth mentioning that reduction of the band gap might cause leakage current during ferroelectric switching. However, leakage current is a common challenge for ferroelectric materials, for example, a large leakage current is observed in ferroelectric Sc-doped w-AlN.^31^ Several factors such as presence of vacancies, domain/grain boundaries, interfaces between the ferroelectric and the electrodes, etc. can cause large leakage current. A detailed understanding of leakage current is beyond the scope of this paper.

The Brillouin zone is sampled with a Γ-centered *k*-point mesh of 6 × 6 × 6 for the structural optimizations and elastic tensor calculations. Considering van der Waals interaction, Born effective charges (*Z*_*ij*_), dielectric constant (ϵ_33_; shown in Supporting Information), and piezoelectric *e*_*ij*_ tensors are obtained from the self-consistent response to finite electric field as implemented using the LCALCEPS = .True. and IBRION = 6 tags using a *k*-point mesh of 3 × 3 × 3. van der Waals interaction is not implemented in VASP for density functional perturbation theory (DFPT) calculations. Therefore, DFPT is employed using the optimized structures obtained with van der Waals interactions, and we calculate the resulting piezoelectric *e*_*ij*_ tensors using *k*-point mesh of 6 × 6 × 6. Elastic *C*_*ij*_ tensors are obtained using the finite difference approach implemented VASP (IBRION = 6 tag) keeping van der Waals interaction using *k*-point mesh of 6 × 6 × 6. To check the convergence of the results with respect to computational setup, the Supporting Information presents *e*_*ij*_, *d*_*ij*_, and *C*_*ij*_ tensors for *k*-point meshes of 3 × 3 × 3, 6 × 6 × 6, and 9 × 9 × 9 and for plane-wave energy cutoffs of 500, 550, and 600 eV, respectively. No substantial alterations in the values have emerged with adjustments in either the *k*-mesh or energy cutoff. This affirms that the outcomes and conclusion presented in this paper from our computational setup are converged with respect to *k*-point sampling and plane-wave cutoff energy.

To simulate the monolayer and trilayer of doped h-BN, we consider 2 × 2 × 1 supercells with a 25 Å vacuum layer between the periodic images along the *c*-direction to avoid their interaction. van der Waals interactions are included for the trilayers. As the slabs are polar, we also include dipole moment correction (IDIPOL = 3 tag in VASP). Allowing relaxation of supercell shape as well as atomic positions for each image, we employ solid-state nudged elastic band (SS-NEB) method^32^ with five images (three images for doped trilayer) to calculate the energy barrier for Al/Ga passing through the h-BN layers. The images are relaxed until the maximum force per atom was no more than 0.01 eV/Å.

## Results and Discussion

Bulk h-BN belongs to centrosymmetric space group *P*6_3_/*mmc*, which consists of planar h-BN monolayers (AA′ stacking, i.e., one h-BN monolayer is 180° rotated with respect to the next layer). Our calculated lattice parameters of h-BN (*a* = *b* = 2.509 Å and *c* = 6.730 Å) with van der Waals interactions are in good agreement with the experimental values (*a* = *b* = 2.504 Å and *c* = 6.660 Å).^33^ We find that AA′ stacking is 9.055 meV/atom lower in energy than AB-type stacking (where N atoms of the layers are on top of each other, but B lie at the center of the hexagon) for bulk h-BN. Therefore, we choose AA′ stacking for further investigation. We also confirm that flat h-BN is 43.377 meV/atom lower in energy than its hypothetical wurtzite structure. On the other hand, both bulk w-AlN and w-GaN have noncentrosymmetric wurtzite structure (space group: *P*6_3_*mc*), where AlN or GaN layers are AA′ stacked, but the layers are buckled along the *c*-direction. Our computed lattice parameters—w-AlN: *a* = *b* = 3.129 Å and *c* = 5.015 Å; w-GaN: *a* = *b* = 3.246 Å and *c* = 5.276 Å—without van der Waals interaction, as the layers are covalently bonded, are also consistent with the experimental values (*a* = 3.110 Å, *c* = 4.980 Å for AlN and *a* = 3.190 Å, *c* = 5.189 Å for GaN).^34^

**Figure 1:**
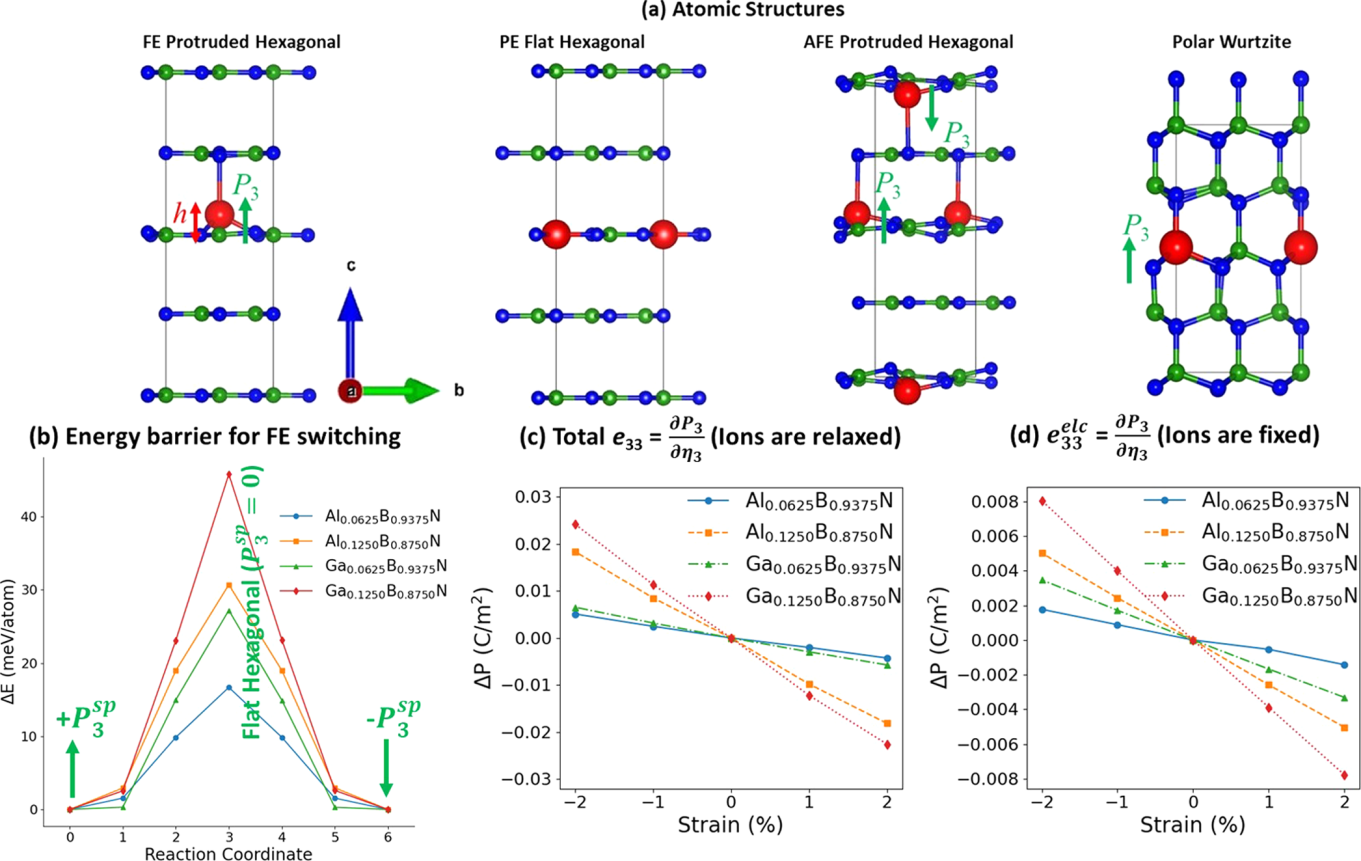
(a) Four atomic structures are considered. We find that doped atoms protrude out of the planar h-BN monolayer, hence resulting in a local dipole moment. For low doping concentration, FE protruded hexagonal structures are energetically more stable than the flat hexagonal or polar wurtzite phase. Also, FE alignment is energetically preferable to AFE. Green, blue, and red balls represent B, N, and Al/Ga atoms, respectively. (b) Energy barrier (Δ*E*) for switching upward to downward FE polarization by displacing the dopant atoms through the planar h-BN monolayer. Therefore, flat h-BN can be considered paraelectric (PE) phase. (c) Piezoelectric constant  is calculated using the finite difference method, where Δ*P = P*_3_(η_3_) – *P*_3_(η_3_ = 0) is the polarization change along the *c*-direction obtained from Berry phase calculation in response to applied strain (η_3_) along the *c*-direction. To calculate (c) total *e*_33_, we relax both ions and electron charge distribution in response to η_3_, whereas ions are kept fixed at their unstrained positions to obtain (d) the electronic part of *e*_33_.

To induce spontaneous polarization and piezoelectric response, we introduce a doping approach by substituting B atoms in h-BN with Al or Ga atoms, resulting in a combination of polar w-AlN and w-GaN within the h-BN lattice structure. We consider low doping concentration below 12.5% to keep the layered structure of bulk h-BN, since above this concentration, a phase transition from the layered structure to the wurtzite structure can occur.^9^ Furthermore, our intention is to maintain the intrinsic van der Waals layered structure of h-BN without fully connecting all of the layers through the dopants. Interestingly, our optimized structures show that Al and Ga atoms protrude out of the otherwise flat h-BN monolayer (Figure 1). The computed thickness *h*, shown in Table 1, of the monolayer measures the extent of the protrusion. This results in a local electric dipole moment along the *c*-direction. Our calculated spontaneous polarization (*P*_3_^sp^) using the modern theory of polarization based on Berry phase^35,36^ with the flat hexagonal structure as the reference paraelectric, is shown in Table 1. Overall, the calculated polarization values are larger than *P*_3_^sp^ of layered FE CuInP_2_S_6_ (4 μC/cm^2^)^16^ and are quite comparable to *P*_3_^sp^ of semicrystalline poly(vinylidene fluoride-trifluoroethylene) (PVDF-TrFE) (8 μC/cm^2^).^16^ However, these are an order of magnitude smaller than *P*_3_^sp^ of Sc-doped w-AlN (∼125 μC/cm^2^).^7,8^ Note that FE-doped h-BN exhibits an order of magnitude larger *P*_3_^sp^ than bilayer h-BN (sliding of the bilayer results in a small *P*_3_^sp^ ∼ 0.68 μC/cm^2^).^12^

**Table 1:** *H*_mix_ is the Mixing Enthalpy of the Protruding Doped h-BN Structures^a^

	*H*_mix_ (eV/fu)	*t*_layer_ (Å)	X–N (in-plane) (Å)	X–N (oop) (Å)	ICOHP_X–N_^in-plane^ (eV)	ICOHP_X–N_^oop^ (eV)	*h* (Å)	*P*_3_^sp^ (μC/cm^2^)	Δ*E* (meV/atom)
h-BN		3.365	1.449	3.365	–10.379	–0.031			
Al_0.0625_B_0.9375_N	0.246	3.242	1.705	2.245	–5.197(−5.577)	–1.780(−0.132)	0.673	7.227	16.684
Al_0.1250_B_0.8750_N	0.426	3.091	1.732	2.224	–5.323(−5.579)	–2.089(−0.141)	0.780	19.943	30.722
Ga_0.0625_B_0.9375_N	0.291	3.292	1.789	2.392	–5.497(−5.972)	–1.184(−0.099)	0.835	7.968	27.213
Ga_0.1250_B_0.8750_N	0.495	3.169	1.825	2.320	–5.731(−6.011)	–1.684(−0.118)	0.992	21.117	45.838

a Average interlayer spacing (*t*_layer_) defined as lattice parameter c divided by the number of layers considered (4). Average X–N (in-plane) and X–N (oop) are bond lengths between metal (X = Al, N) and N in the h-BN plane and out-of-plane N, respectively. ICOHP quantifies the strength of the X–N bonds. ICOHP_X–N_^in-plane^ and ICOHP_X–N_^oop^ represent ICOHP of in-plane and out-of-plane X–N bond, respectively. ICOHP values for the doped flat hexagonal structures are shown in parentheses. *h* is the thickness of a h-BN monolayer with protruding Al/Ga atoms (see Figure 1). *P*_3_^sp^ is the spontaneous polarization. Δ*E* is the energy barrier for FE polarization switching; this also represents the energy difference between protruded FE and flat paraelectric phases (see Figure 1).

We also estimate the energy barrier (Δ*E*) for switching the FE polarization using SS-NEB (see Figure 1b) where we consider flat doped h-BN as a paraelectric reference, and Al/Ga atoms are passing through the h-BN monolayer during the switching process. Here, we assume a uniform polarization switching and ignore the effects of electrodes, surface charges, and domains. Typically, this approach is widely used in DFT-based simulations to estimate the FE switching barrier to predict the possibility of ferroelectricity.^7,8,20,37^ Our calculated Δ*E* values are given in Table 1. Overall, our Δ*E* values are quite comparable to those of FE codoped w-AlN (about 20–45 meV/atom at 25% doping concentration),^8^ Sc-doped w-AlN (e.g., 55.56 meV/atom at 25% Sc and 21.76 meV/atom at 37.50% Sc),^8^ ScN/AlN superlattice (40 meV/atom),^7^ and well-known FE perovskite PbTiO_3_ (40 meV/atom).^38^ The Δ*E* values are lower than that of FE *ABC* materials (e.g., Δ*E* for LiBeP is 240 meV/fu).^28^ Interestingly, although orthorhombic GaFeO_3_ has a high FE switching barrier (1.05 eV/fu), its ferroelectricity has been demonstrated experimentally.^39^

B, Al, and Ga are trivalent cations with ionic radii in IV coordination of 0.11, 0.39, and 0.47 Å, respectively. The presence of larger Al^3+^ and Ga^3+^ cations in the h-BN monolayers can be considered to be the origin of the observed out-of-plane protrusions. Consequently, doping in h-BN decreases the interlayer distance (Table 1). Moreover, the B–N bond length in bulk h-BN (1.449 Å) is shorter than the Al–N (Ga–N) bond of 1.857 Å (1.952 Å) in bulk h-AlN (bulk h-GaN). Table 1 shows that the bond length between Al/Ga and one of the three N atoms in the h-BN plane is much shorter than the bond length along the *c*-direction between Al/Ga and N of another h-BN layer. This suggests that in-plane Al/Ga–N bonds are stronger than out-of-plane Al/Ga–N bonds. To quantify the bond strength, we calculate the integrated crystal orbital Hamilton population (ICOHP), as shown in Table 1. A more negative ICOHP means a stronger covalent bond. Indeed, from the ICOHP, we find that the out-of-plane protrusion of the dopant facilitates a strong covalent bond between Al/Ga and N in the top layer. For example, ICOHP_Al–N_^oop^ for protruded Al_0.125_B_0.875_N composition is −2.089 eV, which is much larger than that of flat structure, for which the computed ICOHP_Al–N_^oop^ is −0.141 eV when Al is in the h-BN plane. As expected from the in-plane and out-of-plane bond length difference, the ICOHP for Al/Ga–N bonds in the plane is quite large (see Table 1).

For protruded doped BN structures, we calculate mixing enthalpy (*H*_mix_) defined as *H*_mix_ = *E*_X_x_B_1–x_N_ – *xE*_w-XN_ – (1 – *x*)*E*_h-BN_, where *x* is the atomic concentration of X (X = Al, Ga) and *E* stands for total energy per formula unit (fu). Our estimated *H*_mix_ values are positive (shown in Table 1), which indicates that nonequilibrium growth processes, such as sputtering, which is already used for doped w-AlN materials, would be an ideal approach to fabricate these materials. Note that our *H*_mix_ values are comparable to that of Sc-doped w-AlN (∼0.3 eV/fu).^5^ Promisingly, B-doped w-AlN has been synthesized by sputtering,^9^ where the B concentration was up to ∼11%. Moreover, sputtering has been employed to grow good-quality h-BN films with thickness from 50 to 120 nm.^40^ Therefore, we propose that sputtering can be used to grow doped h-BN.

To estimate the electromechanical response of the FE-doped h-BN, we calculate piezoelectric stress constant *e*_33_ from the change of the polarization along the *c*-direction, normal to the h-BN plane, as Δ*P* = *P*_3_^sp^(η_3_) – *P*_3_^sp^(η_3_ = 0), in response to a uniaxial strain along the same direction (η_3_). Figure 1c shows a linear relation between Δ*P* and η_3_, and the slope yields *e*_33_. The atoms in the simulation cells are fully relaxed in response to η_3_. Using this slope method, our calculated *e*_33_ values for Al_0.0625_B_0.9375_N, Al_0.1250_B_0.8750_N, Ga_0.0625_B_0.9375_N, and Ga_0.1250_B_0.8750_N are −0.282, −0.912, −0.306, and −1.169 C/m^2^, respectively. Interestingly, all of the FE structures show a negative sign for *e*_33_ as *P*_3_^sp^ decreases in response to the elongation of the *c* lattice parameter (i.e., positive η_3_).

To understand the origin of the negative sign, we decompose *e*_33_ into two terms: *e*_33_^ion^ where the piezoelectric response is only due to ionic displacement in response to η_3_, and *e*_33_^elc^ which comes only from redistribution of electron density in response to η_3_. We calculate *e*_33_^elc^ from the slope of the linear relation between Δ*P* and η_3_ (see Figure 1d), where atoms are kept fixed at their zero strain optimized positions, and only the electron density is optimized in response to η_3_. Our calculated *e*_33_^elc^ (from which *e*_33_^ion^ = *e*_33_ – *e*_33_^elc^) for Al_0.0625_B_0.9375_N, Al_0.1250_B_0.8750_N, Ga_0.0625_B_0.9375_N, and Ga_0.1250_B_0.8750_N are −0.078 (−0.154), −0.251 (−0.661), −0.169 (−0.137), and −0.395 (−0.774) C/m^2^, respectively. We notice that the *e*_33_^ion^ values are much larger than the *e*_33_^elc^. However, both *e*_33_^elc^ and *e*_33_^ion^ have negative signs, which is different from other piezoelectrics that show negative stress constants. For example, FE HfO_2_ (orthorhombic phase with *Pca*2_1_ space group) shows negative *e*_33_ (−1.53 C/m^2^) due to large negative *e*_33_^ion^ (−2.16 C/m^2^) but positive small *e*_33_^elc^ (0.63 C/m^2^).^19^ On the other hand, van der Waals layered FE CuInP_2_S_6_ exhibits a small negative *e*_33_ (−9.7 μC/cm^2^) because of a large negative *e*_33_^elc^ (−10.1 μC/cm^2^) and a small positive *e*_33_^ion^ (0.4 μC/cm^2^).^15^ Overall, our calculated *e*_33_ values are an order of magnitude larger than that of FE CuInP_2_S_6_.^15^

To obtain all of the coefficients of the *e*_*ij*_ tensor, we utilize VASP’s LCALCEPS-tag, in which *e*_*ij*_^elc^ are calculated from the self-consistent response to a finite electric field and *e*_*ij*_^ion^ are obtained from Born effective charges, force constant matrices, and internal strain tensors. In addition, we compute the *e*_*ij*_ tensors using the DFPT method, where we use the same optimized structures but cannot include van der Waals interaction as already described. Our calculated *e*_*ij*_ tensors are given in the Supporting Information. Table 2 shows computed *e*_33_^elc^, *e*_33_^ion^, and *e*_33_. We notice that both the value and sign of the calculated *e*_33_^elc^, *e*_33_^ion^, and *e*_33_ by LCALCEPS and DFPT are quite consistent with those obtained by the slope method (Figure 1c,d). The magnitude of *e*_33_ increases as the doping concentration increases. Overall, our *e*_33_values are quite comparable to that of other known negative piezoelectrics—for example, *e*_33_ of Ga_0.125_B_0.875_N (−1.164 C/m^2^) is comparable to that of NaZnSb (−1.23 C/m^2^).^19^

**Table 2:** Ionic (*e*_33_^ion^) and Electronic (*e*_33_^elc^) Parts of Total *e*_33_ Obtained from the Self-Consistent Response to a Finite Electric Field ε (LCALCEPS-tag in VASP) are in C/m^2^ Unit^a^

	*e*_33_^ion^	*e*_33_^ion^(B)	*e*_33_^ion^(N)	*e*_33_^ion^(M)	*e*_33_^elc^	*e*_33_	*C*_33_	*d*_33_
Al_0.0625_B_0.9375_N	–0.152(−0.158)	–0.138	–0.029	0.016	–0.076(−0.077)	–0.228(−0.235)	43.980	–5.154(−5.309)
Al_0.1250_B_0.8750_N	–0.673(−0.710)	–0.412	–0.190	–0.051	–0.241(−0.242)	–0.914(−0.952)	56.907	–16.016(−16.634)
Ga_0.0625_B_0.9375_N	–0.135(−0.142)	–0.092	–0.021	–0.016	–0.157(−0.158)	–0.292(−0.300)	39.857	–7.315(−7.506)
Ga_0.1250_B_0.8750_N	–0.776(−0.804)	–0.357	–0.209	–0.202	–0.388(−0.392)	–1.164(−1.196)	47.832	–24.214(−24.883)

a The piezoelectric constants obtained from DFPT calculations using the optimized structures considering van der Waals interaction are shown in parentheses. Elastic constant *C*_33_ and longitudinal piezoelectric strain constant *d*_33_ are in GPa and pC/N unit, respectively. Note that 1 C/N is equal to 1 m/V.

To understand the origin of large negative *e*_33_^ion^, we obtain the contribution from each atomic species by decomposing *e*_33_^ion^: *e*_33_^ion^ = ∑_*s*_*e*_33_^ion^(*s*), where *s* runs over all of the atomic species in the supercells (i.e., B, N, Al/Ga). *e*_33_^ion^(*s*) is expressed as , where *i* runs over all of the atoms of a particular species in the supercells, *c* is the lattice parameter, |*e*| is the magnitude of an electron’s charge, *Z*_33_(*i*) is the 33 component of the Born effective charges (BEC) of *i*th atom, *u*_3_ is the fractional position of an atom along *c*-direction, and Ω is the volume of the supercells. Surprisingly, we find that contributions from B and N atoms dominate in *e*_33_^ion^ (Table 2), suggesting that negative piezoelectricity mainly originates from the ionic movement of B and N, and the dopant atoms (Al or Ga) are responsible for breaking the inversion symmetry of bulk h-BN to induce piezoelectricity. Interestingly, we also observe that almost half of the B or N atoms show a positive , i.e., they move in the positive *c*-direction in response to the elongation of c lattice parameter, for which η_3_ is positive, while the other B/N atoms move in the negative *c*-direction and show negative . This can be attributed to the role of the Al/Ga atom as a linker connecting two h-BN planes. The *Z*_33_ and the  of each atom in the supercells are provided in the Supporting Information.

We also obtain the piezoelectric strain tensor *d*_*ij*_ from *e*_*ij*_ and elastic tensor *C*_*ij*_ using the relation: . Typically, *d*_33_ is the important piezoelectric constant for applications, such as resonators or actuators. We find that FE-doped h-BN exhibits a surprisingly large value, *d*_33_ (from −5.154 to 24.214 pC/N), Table 2. This is about 5 times larger than that of commercially used w-AlN (*d*_33_ ∼ 5 pC/N), while w-GaN has a low *d*_33_ of ∼3 pC/N.^21^ We notice that both *e*_33_ and *d*_33_ exhibit a nonlinear increase with the doping concentration. Nonlinear enhancement of the piezoelectric response has also been observed in Sc-doped w-AlN.^22^ Unlike w-AlN, the computed *d*_33_ in doped h-BN shows a negative sign because of the negative *e*_33_, suggesting that these doped structures would shrink (expend) in response to an electric field along the positive (negative) *z*-direction. Our doped h-BN structures are van der Waals layered materials and hence possess a quite low *C*_33_ (see Table 2). This facilitates the large *d*_33_, which is comparable to that of Sc-doped w-AlN, for which *d*_33_ ∼ 25 pC/N at around 27% Sc doping^22^ and also that of FE polymer PVDF-TrFE, for which *d*_33_ = −25 pC/N.^16^ Therefore, these doped h-BN can be a good material for piezoelectric applications in energy harvesting or as actuators.

Recently, it has been suggested that piezoelectric materials that exhibit the same sign for transverse expansion, with coefficients *d*_31_ and *d*_32_, representing a horizontal expansion/contraction of the material in response to an applied electric field along the *c*-direction and longitudinal coefficient *d*_33_, which represents vertical expansion/contraction of the material due to an external electric field along the *c*-direction can show an electric auxetic effect.^19^ In such materials, negative (positive) *d*_31_, *d*_32_, and *d*_33_ result in a contraction (expansion) in all dimensions, in response to an applied electric field. Allowing structural deformations using electric fields, the piezoelectric auxetic effect can enable the development of highly sensitive sensors and transducers for applications such as pressure sensors, accelerometers, and strain gauges. This effect can also be harnessed to create smart actuators and nanosystems. The combination of the piezoelectric response and the auxetic effect allows for precise control and manipulation of mechanical strain, enabling the development of advanced microactuators, microfluidic devices, and nanoelectromechanical systems.^19,41,42^ These systems find applications in fields such as robotics, biomedical devices, and precision engineering. Note that most existing piezoresistances do not show this effect. For example, w-AlN has positive *d*_33_ but negative *d*_31_ (−2.17 pC/N). However, the FE orthorhombic phase of HfO_2_ shows an electric auxetic effect, where *d*_31_ (−1.25 pm/V), *d*_32_ (−1.84 pm/V), and *d*_33_ (−2.59 pm/V) have a negative sign.^19^Table 3 shows our calculated transverse piezoelectric coefficients, *e*_31_, *e*_32_, *d*_31_, and *d*_32_. All of the FE-doped h-BN structures have negative *e*_31_ and *e*_32_ originating from the ionic contribution, as we observe large negative *e*_31_^ion^ and *e*_32_^ion^ but small positive *e*_31_^elc^ and *e*_32_^elc^. We find that all of the structures show negative *d*_31_, *d*_32_, and *d*_33_ expect Al_0.1250_B_0.8750_N, which can exhibit quite small *d*_31_ (although *d*_31_ calculated from DFPT has a small negative value). Hence, these doped h-BN structures can show an electric auxetic effect, which would be important for nanoelectromechanical systems, e.g., smart actuators, sensors, and energy harvesters. Note that *d*_31_ and *d*_32_ are quite small compared to *d*_33_. Unlike interlayer out-of-plane weak van der Waals interaction, the dopant atoms have strong in-plane covalent bonds. As a result, the structures have significantly larger in-plane elastic coefficients (e.g., *C*_11_ = 605.731 GPa for Al_0.1250_B_0.8750_N; see the Supporting Information for *C*_*ij*_ tensors) than *C*_33_ (56.907 GPa; see Table 2).

**Table 3:** Ionic (*e*_31_^ion^ and *e*_32_^ion^) and Electronic (*e*_31_^elc^ and *e*_32_^elc^) Parts of Transverse Piezoelectric Stress Coefficients *e*_31_ and *e*_32_ in the C/m^2^ Unit are Calculated by the Self-Consistent Response to a Finite Electric Field ε (LCALCEPS-tag in VASP)^a^

	*e*_31_^ion^	*e*_31_^elc^	*e*_31_	*e*_32_^ion^	*e*_32_^elc^	*e*_32_	*d*_31_	*d*_32_
Al_0.0625_B_0.9375_N	–0.230 (−0.238)	0.014 (0.014)	–0.216 (−0.224)	–0.230 (−0.238)	0.014 (0.014)	–0.216 (−0.224)	–0.214 (−0.221)	–0.214 (−0.221)
Al_0.1250_B_0.8750_N	–0.321 (−0.37975)	0.067 (0.06835)	–0.254 (−0.311)	–0.423 (−0.476)	0.055 (0.057)	–0.367 (−0.419)	0.064 (−0.010)	–0.162 (−0.223)
Ga_0.0625_B_0.9375_N	–0.258 (−0.263)	0.056 (0.054)	–0.202 (−0.210)	–0.258 (−0.263)	0.056 (0.054)	–0.202 (−0.210)	–0.203 (−0.211)	–0.203 (−0.211)
Ga_0.1250_B_0.8750_N	–0.491 (−0.505)	0.124 (0.125)	–0.368 (−0.380)	–0.621 (−0.619)	0.119 (0.121)	–0.502 (−0.499)	–0.040 (−0.058)	–0.246 (−0.224)

a The piezoelectric coefficients obtained from DFPT calculations are shown in parentheses. Transverse piezoelectric strain coefficients *d*_31_ and *d*_31_ are in pC/N unit.

**Figure 2:**
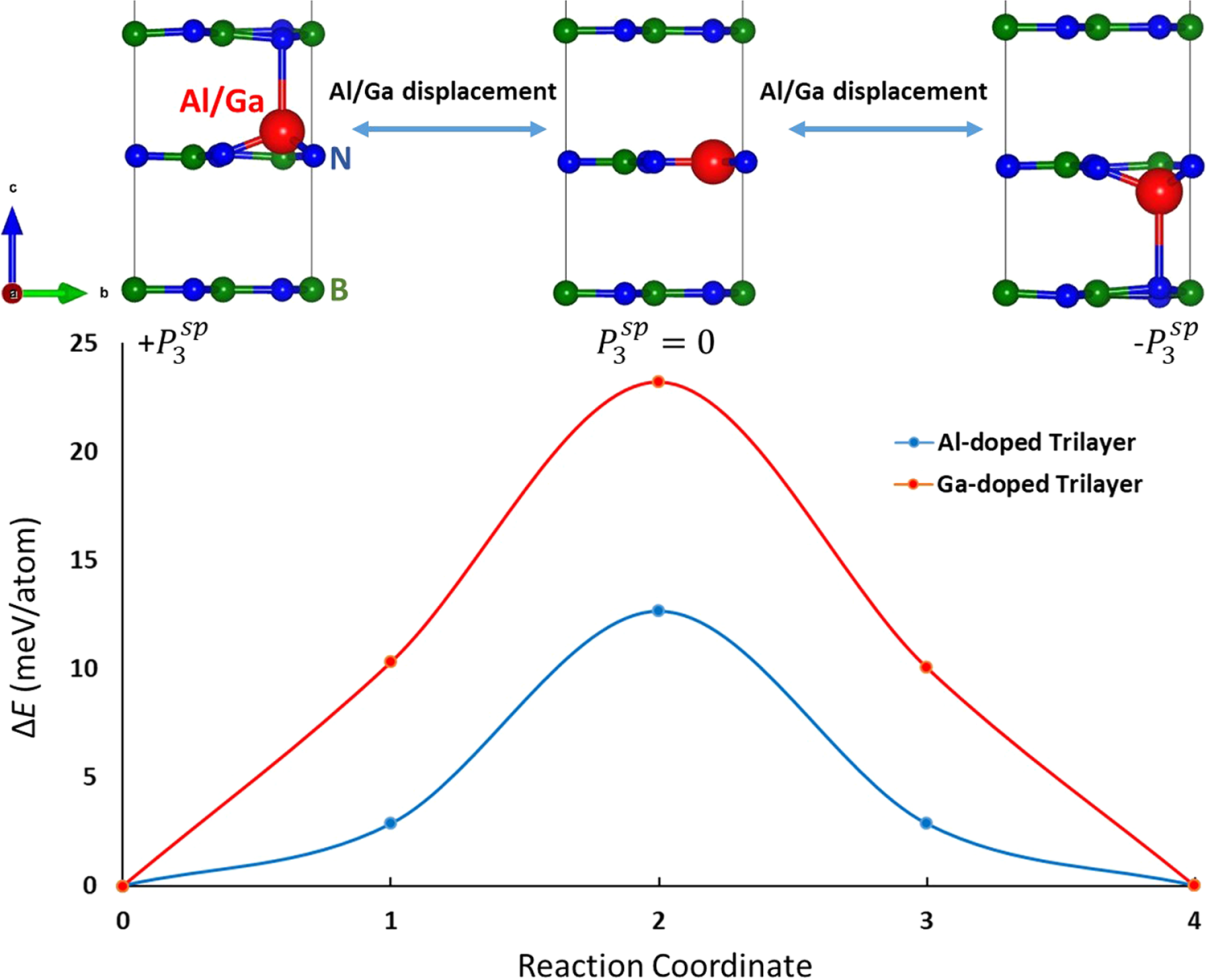
Energy barrier obtained from SS-NEB calculations for FE polarization switching in doped h-BN trilayer, where the Al/Ga atom passes through the h-BN layer. We consider flat phase as a paraelectric reference and also notice that the flat phase is higher in energy compared to polar protruded phase.

To this end, it is interesting to know if the protruded polar phase remains stable down to a few layer thickness as typically there is a critical thickness for ferroelectricity in perovskites due to the depolarization field. Our DFT-optimized structures show that freestanding doped h-BN monolayer (1 B atom is replaced by 1 Al/Ga in a 2 *×* 2 × 1 monolayer supercell) is one atomic flat like pristine h-BN monolayer and therefore will not display out-of-plane polarization. Note that there 2D materials where doped atoms are protruded from the plane such as Mn-doped graphene.^43^ However, we find that in an h-BN trilayer Al/Ga doping prefers the protruded phase to the flat structure, and hence these dopants induce a spontaneous polarization along the *c*-direction. Here, we consider a 2 *×* 2 × 1 trilayer supercell, and 1 B atom is replaced by 1 Al/Ga at the center layer (see Figure 2) of the trilayer to keep the up and down polarization states degenerate in terms of energy. The doping concentration is 4.167%. Based on the modern theory of polarization, our calculated *P*_3_^sp^ for Al (Ga) doped trilayer is 5.186 μC/m^2^ (4.216 μC/m^2^); here, the thickness of the trilayer is used to obtain the three-dimensional (3D) unit of C/m^2^. Interestingly, the *P*_3_^sp^ of the trilayer is slightly lower than that of our doped bulk h-BN, Table 1, because the protrusion height *h* of the trilayers (0.609 Å for Al and 0.757 Å for Ga) is smaller compared to their bulk counterparts. This is also evident in the larger interlayer Al/Ga–N bond lengths (2.315 Å for Al–N and 2.530 Å for Ga–N), resulting in lower ICOHP_Al/Ga–N_^oop^ values (−1.535 eV for Al–N and −0.829 eV for Ga–N), compared to their bulk counterparts, as shown in Table 1. It is worth noting that the ICOHP_Al/Ga–N_^oop^ values (−0.131 eV for Al–N and −0.110 eV for Ga–N) of the trilayer flat structure are considerably smaller than those of the protruded structure, indicating that the protrusion facilitates the formation of strong interlayer Al/Ga–N bonds. Moreover, the computed FE switching energy barrier (Δ*E*) for the Al (Ga) doped trilayer is 12.629 meV/atom (23.200 meV/atom), which is also essentially the same as that of the bulk counterpart (see Δ*E* in Table 1). The Δ*E* values for the doped trilayers are also quite comparable to that of other 2D FE materials, e.g., 31.202 meV/atom for VOCl_2_ monolayer^44^ and 60 meV per unit cell for In_2_Se_3_ monolayer.^45^ Therefore, we propose that low-concentration doping of Al/Ga in h-BN can realize ferroelectricity down to the nanoscale, which would offer significant benefits to FE memory technology. Of course, the interaction between such ultrathin FE films and substrate or electrodes, effect of epitaxial strain, FE domain dynamics, etc. need to be understood. Note that epitaxial tensile (compression) strain can reduce (increase) the FE switching barrier with enhanced (reduced) piezo-response.^7,37^ However, these are beyond the scope of this article.

## Conclusions

We predict that low-concentration doping of Al/Ga at the B site in h-BN breaks its inversion symmetry. Because Al/Ga atoms are protruded out of the h-BN plane, which induces local electric dipole moments along the *c*-axis. The induced *P*_3_^sp^ values are comparable to those of other known FE materials. Moreover, *P*_3_^sp^ can be switched by passing Al/Ga through the h-BN plane. We also show that ferroelectricity can persist in these doped h-BN down to the trilayer, allowing significant thickness reduction. Note that bulk h-BN is a nonpiezoelectric material due to the inversion symmetry. However, surprisingly, we find a huge negative piezoelectric coefficient *d*_33_ in these doped h-BN, e.g., −24.214 pC/N for Ga_0.125_B_0.875_N, which is about 5 times larger than that of pure w-AlN and comparable to Sc-AlN, because of their quite low *C*_33_ in the van der Waals layered structure. Interestingly, these doped h-BN materials can also exhibit an electric auxetic effect. As h-BN layers are widely used in 2D materials-based devices as substrate or dielectric layer for SiO_2_/Si substrates, our findings can broaden its applications to FE memories or piezoelectric energy harvesting.

## Supporting Information Available

Change of the lattice parameters during the ferroelectric switching; electronic density of states (DOS); macroscopic dielectric constant ϵ_33_; optimized structure, *e*_*ij*_, *C*_*ij*_, and *d*_*ij*_ of protruded Al_0.0556_B_0.9444_N structure (3 × 3 × 2 supercell), protruded Ga_0.0556_B_0.9444_N structure (3 × 3 × 2 supercell), protruded Al_0.1250_B_0.8750_N structure (2 × 2 × 4 supercell), protruded Ga_0.1250_B_0.8750_N structure (2 × 2 × 4 supercell), protruded Al_0.0625_B_0.9375_N structure (2 × 2 × 2 supercell), protruded Al_0.1250_B_0.8750_N structure (2 × 2 × 2 supercell), protruded Ga_0.0625_B_0.9375_N structure (2 × 2 × 2 supercell), and protruded Ga_0.1250_B_0.8750_N structure (2 × 2 × 2 supercell); convergence of *e*_*ij*_^ion^, *e*_*ij*_^elc^, *C*_*ij*_, and *d*_*ij*_ in terms of *k*-point meshes; and plane-wave energy cutoffs (ENCUT) (PDF)

## References

[ref1] Kim M.-K.; Lee J.-S. Ferroelectric Analog Synaptic Transistors. Nano Lett. 2019, 19, 2044–2050. 10.1021/acs.nanolett.9b00180. 30698976

[ref2] Oh S.; Hwang H.; Yoo I. K. Ferroelectric Materials for Neuromorphic Computing. APL Mater. 2019, 7, 091109 10.1063/1.5108562.

[ref3] Soliman M.; Maity K.; Gloppe A.; Mahmoudi A.; Ouerghi A.; Doudin B.; Kundys B.; Dayen J.-F. Photoferroelectric All-van-der-Waals Heterostructure for Multimode Neuromorphic Ferroelectric Transistors. ACS Appl. Mater. Interfaces 2023, 15, 15732–15744. 10.1021/acsami.3c00092. 36919904 PMC10375436

[ref4] Junquera J.; Ghosez P. Critical Thickness for Ferroelectricity in Perovskite Ultrathin Films. Nature 2003, 422, 506 10.1038/nature01501. 12673246

[ref5] Zhang S.; Holec D.; Fu W. Y.; Humphreys C. J.; Moram M. A. Tunable Optoelectronic and Ferroelectric Properties in Sc-based III-nitrides. J. Appl. Phys. 2013, 114, 133510 10.1063/1.4824179.

[ref6] Fichtner S.; Wolff N.; Lofink F.; Kienle L.; Wagner B. AlScN: A III-V Semiconductor Based Ferroelectric. J. Appl. Phys. 2019, 125, 114103 10.1063/1.5084945.

[ref7] Noor-A-Alam M.; Z Olszewski O.; Nolan M. Ferroelectricity and Large Piezoelectric Response of AlN/ScN Superlattice. ACS Appl. Mater. Interfaces 2019, 11, 20482–20490. 10.1021/acsami.8b22602. 31074260

[ref8] Noor-A-Alam M.; Olszewski O. Z.; Campanella H.; Nolan M. Large Piezoelectric Response and Ferroelectricity in Li and V/Nb/Ta Co-Doped w-AlN. ACS Appl. Mater. Interfaces 2021, 13, 944–954. 10.1021/acsami.0c19620. 33382599

[ref9] Hayden J.; Hossain M. D.; Xiong Y.; Ferri K.; Zhu W.; Imperatore M. V.; Giebink N.; Trolier-McKinstry S.; Dabo I.; Maria J.-P. Ferroelectricity in Boron-substituted Aluminum Nitride Thin Films. Phys. Rev. Mater. 2021, 5, 044412 10.1103/PhysRevMaterials.5.044412.

[ref10] Molaei M. J.; Younas M.; Rezakazemi M. A Comprehensive Review on Recent Advances in Two-Dimensional (2D) Hexagonal Boron Nitride. ACS Appl. Electron. Mater. 2021, 3, 5165–5187. 10.1021/acsaelm.1c00720.

[ref11] Quezada-Lopez E. A.; Joucken F.; Chen H.; Lara A.; Davenport J. L.; Hellier K.; Taniguchi T.; Watanabe K.; Carter S.; Ramirez A. P.; Velasco J. J. Persistent and Reversible Electrostatic Control of Doping in Graphene/Hexagonal Boron Nitride Heterostructures. J. Appl. Phys. 2020, 127, 044303 10.1063/1.5127770.

[ref12] Yasuda K.; Wang X.; Watanabe K.; Taniguchi T.; Jarillo-Herrero P. Stacking-engineered Ferroelectricity in Bilayer Boron Nitride. Science 2021, 372, 1458–1462. 10.1126/science.abd3230. 34045323

[ref13] Zhang Q.; Zuo S.; Chen P.; Pan C. Piezotronics in Two-dimensional Materials. InfoMat 2021, 3, 987–1007. 10.1002/inf2.12220.

[ref14] Zhu K.; Pazos S.; Aguirre F.; Shen Y.; Yuan Y.; Zheng W.; Alharbi O.; Villena M. A.; Fang B.; Li X.; Milozzi A.; Farronato M.; Muñoz-Rojo M.; Wang T.; Li R.; Fariborzi H.; Roldan J. B.; Benstetter G.; Zhang X.; Alshareef H. N.; Grasser T.; Wu H.; Ielmini D.; Lanza M. Hybrid 2D–CMOS microchips for memristive applications. Nature 2023, 618, 57–62. 10.1038/s41586-023-05973-1. 36972685 PMC10232361

[ref15] Qi Y.; Rappe A. M. Widespread Negative Longitudinal Piezoelectric Responses in Ferroelectric Crystals with Layered Structures. Phys. Rev. Lett. 2021, 126, 217601 10.1103/PhysRevLett.126.217601. 34114845

[ref16] You L.; Zhang Y.; Zhou S.; Chaturvedi A.; Morris S. A.; Liu F.; Chang L.; Ichinose D.; Funakubo H.; Hu W.; Wu T.; Liu Z.; Dong S.; Wang J. Origin of Giant Negative Piezoelectricity in a Layered van der Waals Ferroelectric. Sci. Adv. 2019, 5, eaav3780 10.1126/sciadv.aav3780. 31016240 PMC6474765

[ref17] Liu S.; Cohen R. E. Origin of Negative Longitudinal Piezoelectric Effect. Phys. Rev. Lett. 2017, 119, 207601 10.1103/PhysRevLett.119.207601. 29219344

[ref18] Noor-A-Alam M.; Nolan M. Negative Piezoelectric Coefficient in Ferromagnetic 1H-LaBr2Monolayer. ACS Appl. Electron. Mater. 2022, 4, 850–855. 10.1021/acsaelm.1c01214. 35224502 PMC8867721

[ref19] Liu J.; Liu S.; Yang J.-Y.; Liu L. Electric Auxetic Effect in Piezoelectrics. Phys. Rev. Lett. 2020, 125, 197601 10.1103/PhysRevLett.125.197601. 33216563

[ref20] Jiang W.; Liu C.; Ma X.; Yu X.; Hu S.; Li X.; Burton L. A.; Liu Y.; Chen Y.; Guo P.; Kong X.; Bellaiche L.; Ren W. Anomalous Ferroelectricity and Double-negative Effects in Bilayer Hexagonal Boron Nitride. Phys. Rev. B 2022, 106, 054104 10.1103/PhysRevB.106.054104.

[ref21] Lueng C. M.; Chan H. L. W.; Surya C.; Choy C. L. Piezoelectric Coefficient of Aluminum Nitride and Gallium Nitirde. J. Appl. Phys. 2000, 88, 5360–5363. 10.1063/1.1317244.

[ref22] Akiyama M.; Kamohara T.; Kano K.; Teshigahara A.; Takeuchi Y.; Kawahara N. Enhancement of Piezoelectric Response in Scandium Aluminum Nitride Alloy Thin Films Prepared by Dual Reactive Cosputtering. Adv. Mater. 2009, 21, 593–596. 10.1002/adma.200802611. 21161988

[ref23] Perdew J. P.; Burke K.; Ernzerhof M. Generalized Gradient Approximation Made Simple. Phys. Rev. Lett. 1996, 77, 3865 10.1103/PhysRevLett.77.3865. 10062328

[ref24] Kresse G.; Furthmüller J. Efficient Iterative Schemes for ab initio Total-Energy Calculations Using a Plane-wave Basis Set. Phys. Rev. B 1996, 54, 11169 10.1103/PhysRevB.54.11169. 9984901

[ref25] Kresse G.; Joubert D. From Ultrasoft Pseudopotentials to the Projector Augmented-wave Method. Phys. Rev. B 1999, 59, 1758 10.1103/PhysRevB.59.1758.

[ref26] Grimme S.; Antony J.; Ehrlich S.; Krieg H. A Consistent and Accurate ab initio Parametrization of Density Functional Dispersion Correction (DFT-D) for the 94 Elements H-Pu. J. Chem. Phys. 2010, 132, 154104 10.1063/1.3382344. 20423165

[ref27] Cassabois G.; Valvin P.; Gil B. Hexagonal boron nitride is an indirect bandgap semiconductor. Nat. Photonics 2016, 10, 262–266. 10.1038/nphoton.2015.277.

[ref28] Bennett J. W.; Garrity K. F.; Rabe K. M.; Vanderbilt D. Hexagonal ABC Semiconductors as Ferroelectrics. Phys. Rev. Lett. 2012, 109, 167602 10.1103/PhysRevLett.109.167602. 23215130

[ref29] Ding W.; Zhu J.; Wang Z.; Gao Y.; Xiao D.; Gu Y.; Zhang Z.; Zhu W. Prediction of intrinsic two-dimensional ferroelectrics in In_2_Se_3_ and other III_2_-VI_3_ van der Waals materials. Nat. Commun. 2017, 8, 14956 10.1038/ncomms14956. 28387225 PMC5385629

[ref30] Parmar V. B.; Raval D.; Gupta S. K.; Gajjar P.; Vora A. BaTiO3 perovskite for optoelectronics application: A DFT study. In Materials Today: Proceedings; Elsevier, 2023.

[ref31] Kataoka J.; Tsai S.-L.; Hoshii T.; Wakabayashi H.; Tsutsui K.; Kakushima K. A possible origin of the large leakage current in ferroelectric Al_1–*x*_Sc_*x*_N films. Jpn. J. Appl. Phys. 2021, 60, 030907 10.35848/1347-4065/abe644.

[ref32] Sheppard D.; Xiao P.; Chemelewski W.; Johnson D. D.; Henkelman G. A Generalized Solid-state Nudged Elastic Band Method. J. Chem. Phys. 2012, 136, 074103 10.1063/1.3684549. 22360232

[ref33] Solozhenko V.; Will G.; Elf F. Isothermal Compression of Hexagonal Graphite-like Boron Nitride up to 12 GPa. Solid State Commun. 1995, 96, 1–3. 10.1016/0038-1098(95)00381-9.

[ref34] Schulz H.; Thiemann K. Crystal Structure Refinement of AlN and GaN. Solid State Commun. 1977, 23, 815–819. 10.1016/0038-1098(77)90959-0.

[ref35] King-Smith R. D.; Vanderbilt D. Theory of Polarization of Crystalline Solids. Phys. Rev. B 1993, 47, 1651–1654. 10.1103/PhysRevB.47.1651. 10006189

[ref36] Vanderbilt D.; King-Smith R. D. Electric Polarization as a Bulk Quantity and its Relation to Surface Charge. Phys. Rev. B 1993, 48, 4442–4455. 10.1103/PhysRevB.48.4442. 10008920

[ref37] Moriwake H.; Konishi A.; Ogawa T.; Fujimura K.; Fisher C. A. J.; Kuwabara A.; Shimizu T.; Yasui S.; Itoh M. Ferroelectricity in Wurtzite Structure Simple Chalcogenide. Appl. Phys. Lett. 2014, 104, 242909 10.1063/1.4884596.

[ref38] Yuk S. F.; Krishna Chaitanya P.; Serge M N.; Markus E.; Ying Wai L.; Valentino R C. Towards an Accurate Description of Perovskite Ferroelectrics: Exchange and Correlation Effects. Sci. Rep. 2017, 7, 43482 10.1038/srep43482. 28256544 PMC5335310

[ref39] Song S.; Jang H. M.; Lee N.-S.; Son J. Y.; Gupta R.; Garg A.; Ratanapreechachai J.; Scott J. F. Ferroelectric Polarization Switching with a Remarkably High Activation Energy in Orthorhombic GaFeO3 Thin Films. NPG Asia Mater. 2016, 8, e242 10.1038/am.2016.3.

[ref40] Li Q.; Wang M.; Bai Y.; Zhang Q.; Zhang H.; Tian Z.; Guo Y.; Zhu J.; Liu Y.; Yun F.; Wang T.; Hao Y. Two-Inch Wafer-Scale Exfoliation of Hexagonal Boron Nitride Films Fabricated by RF-Sputtering. Adv. Funct. Mater. 2022, 32, 2206094 10.1002/adfm.202206094.

[ref41] Roy Chowdhury A.; Saurabh N.; Kiran R.; Patel S. Effect of Porous Auxetic Structures on Low-frequency Piezoelectric Energy Harvesting Systems: a Finite Element Study. Appl. Phys. A: Mater. Sci. Process. 2022, 128, 62 10.1007/s00339-021-05199-w.

[ref42] Eghbali P.; Younesian D.; Moayedizadeh A.; Ranjbar M. Study in Circular Auxetic Structures for Efficiency Enhancement in Piezoelectric Vibration Energy Harvesting. Sci. Rep. 2020, 10, 16338 10.1038/s41598-020-73425-1. 33004956 PMC7531003

[ref43] Noor-A-Alam M.; Ullah H.; Shin Y.-H. Switchable Polarization in Mn Embedded Graphene. Sci. Rep. 2018, 8, 4538 10.1038/s41598-018-22583-4. 29540731 PMC5852129

[ref44] Noor-A-Alam M.; Nolan M. Large Piezoelectric Response in Ferroelectric/Multiferroelectric Metal Oxyhalide MOX2 (M = Ti, V and X = F, Cl and Br) Monolayers. Nanoscale 2022, 14, 11676–11683. 10.1039/D2NR02761E. 35912821

[ref45] Tang Z.; Dai M.; Chen Y.; He Q.; Luo X.; Zheng Y. Strain Engineering the Ferroelectric Polarization and Optical Absorption in the FE β-In2Se3Monolayer. J. Phys. Chem. C 2022, 126, 10181–10189. 10.1021/acs.jpcc.2c01352.

