# Supplementary material for: Engineering Ferroelectricity and Large Piezoelectricity in h-BN
Source: ACS Appl Mater Interfaces. 2023 Aug 31;15(36):42737–45. doi: 10.1021/acsami.3c07744 (PMC10510043; doi:10.1021/acsami.3c07744)
Supplement: Supplementary file 1 — am3c07744_si_001.pdf [file am3c07744_si_001.pdf]

Supporting Information: Engineering Ferroelectricity and Large Piezoelectricity in *h*-BN

Mohammad Noor-A-Alam and Michael Nolan**

Tyndall National Institute, University College Cork, Lee Maltings, Dyke Parade, Cork T12 R5CP, Ireland

E-mail: mda.alam@tyndall.ie;michael.nolan@tyndall.ie

Change of the lattice parameters during the ferroelectric switching:

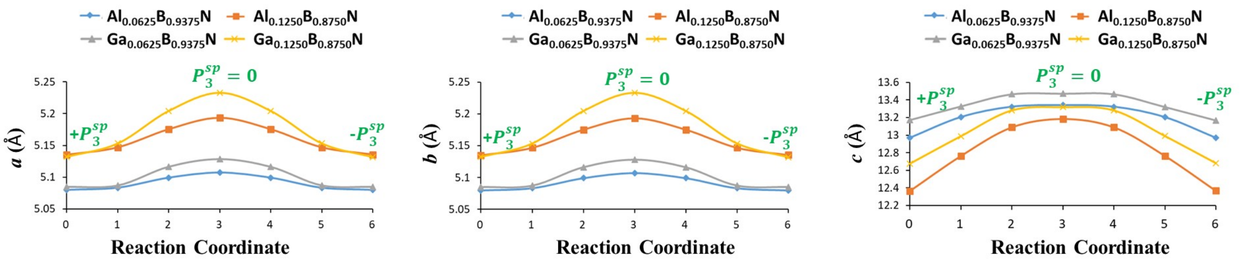

Figure S1: Change of the lattice parameters (a, b, and c in Å of $2 \times 2 \times 2$ supercell) during the ferroelectric polarization switching for the doped bulk h-BN. Our calculated $a = b$ and c of pure bulk h-BN unitcell including van der Waals interaction (DFT-D3) are 2.509 Å and 6.730 Å, respectively. The values of a and b are nearly equal, suggesting minimal structural distortion due to doping within the lattice. We observe an expansion in both the horizontal and vertical directions as the structures transition from the ferroelectric protruded phase with a polarization of $\pm P_3^{sp}$ to the paraelectric phase where P_3^{sp} equals zero. The expansion is more pronounced in the Ga-doped cases due to the larger size of the Ga^{3+} ion compared to the Al^{3+} ion.

Electronic Density of States (DOS):

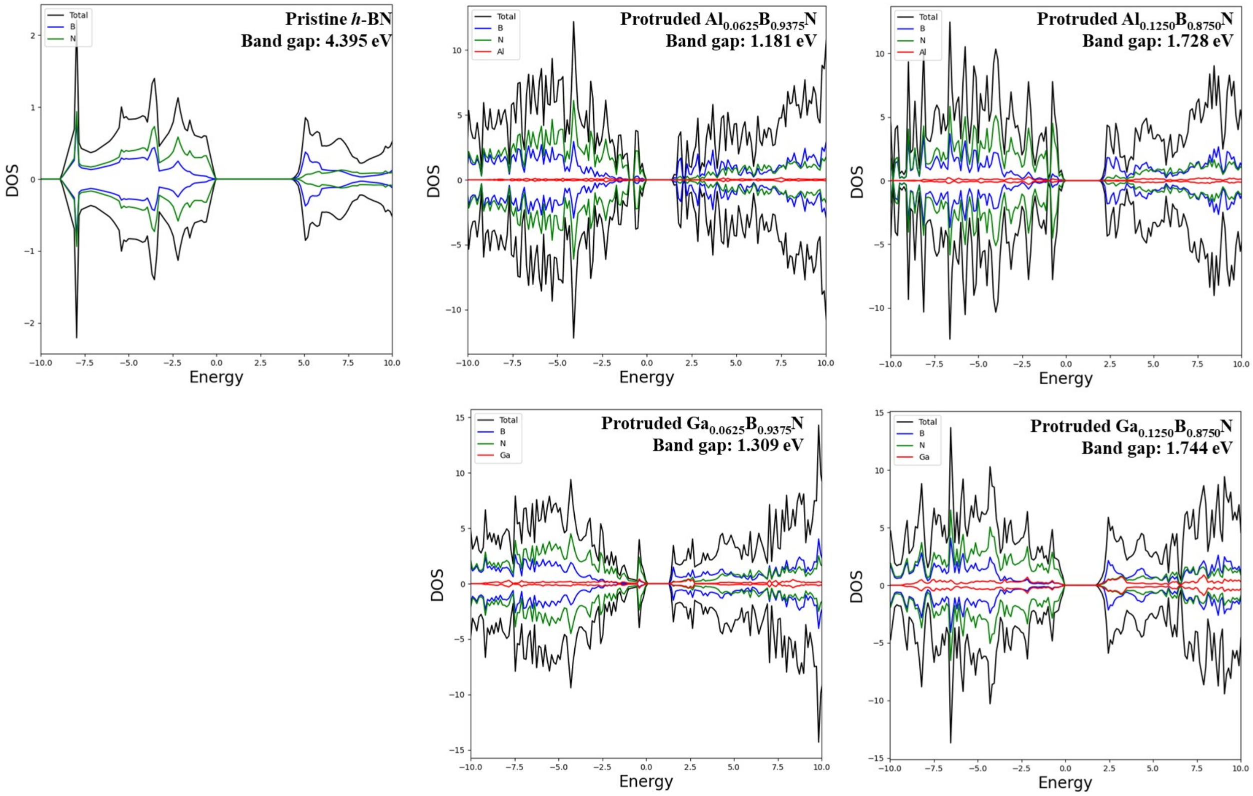

Figure S2: Electronic total DOS (states/eV) and element projected DOS (states/eV) of pristine as well as doped *h*-BN calculated at the GGA-PBE level. The Fermi level is set to 0 eV. Spin-up and spin-down are differentiated with opposite signs in DOS. $2 \times 2 \times 2$ supercell is used for the doped structures. We see that both Al and Ga doping reduce the large band gap of pristine bulk *h*-BN. The Fermi level is primarily influenced by contributions from N atoms, while contributions from B atoms take precedence around the minimum of the conduction band. Al/Ga atoms also contribute at the Fermi level and at the minimum of the conduction band. It's worth noting that the GGA-PBE method tends to underestimate the band gap, implying that experimental results are likely to yield a larger band gap.

The macroscopic dielectric Constant ϵ_{33}:

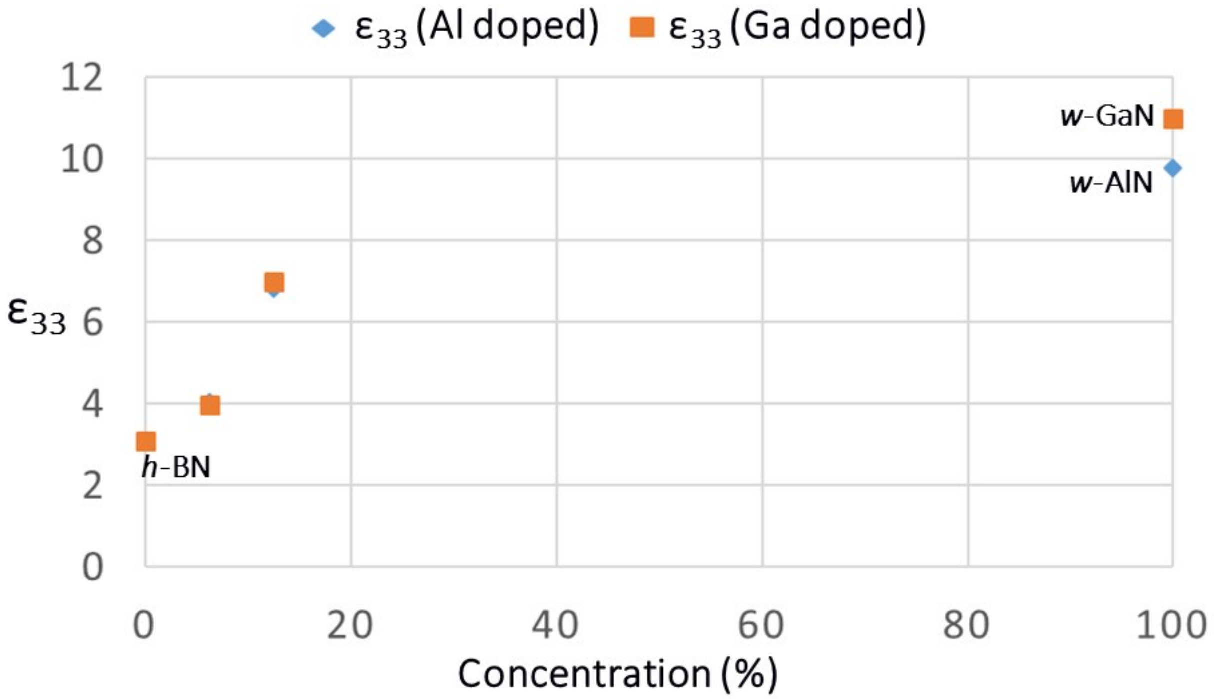

Figure S3: We estimate the zz component of the macroscopic dielectric constant (ε_{33}) using LCALCEPS tag in VASP. Here we add high-frequency dielectric constant including local field effects or electronic contribution $\varepsilon_{33}^{\infty}$ and the static dielectric constant or ionic contribution ε_{33}^{0} to get the total ε_{33}. $2 \times 2 \times 2$ supercell is used for the doped structures. We see that ε_{33} increase as the doping concentration increases.

Protruded $Al_{0.0556}B_{0.9444}N$ Structure ($3 \times 3 \times 2$ supercell):

Optimized Structure:

FE-$Al_{0.0556}B_{0.9444}N$

1.00000000000000

7.5823954761619587 0.0000000000000000 0.0000000000000000

-3.7911977380809794 6.5665471038964638 -0.0000000000000000

0.0000000000000000 0.0000000000000000 13.2589009692992441

B N Al

34 36 2

Direct

0.1139198597705552 0.2276094710490933 -0.0004537992162181

0.4470229446118740 0.2276094710490933 -0.0004537992162181

0.7777777777777786 0.2222222222222214 -0.0007880646034964

0.1139198597705552 0.5529770553881265 -0.0004537992162181

0.4444444444444429 0.5555555555555571 -0.0172449664508108

0.7723905289509065 0.5529770553881265 -0.0004537992162181

0.4470229446118740 0.8860801402294449 -0.0004537992162181

0.7723905289509065 0.8860801402294449 -0.0004537992162181

0.1057117288478388 0.2196859659666706 0.4998022745545507

0.4473075704521675 0.2196859659666706 0.4998022745545507

0.7777777777777786 0.2222222222222214 0.4835834615293184

0.1057117288478388 0.5526924295478325 0.4998022745545507

0.7803140340333292 0.5526924295478325 0.4998022745545507

0.1111111111111143 0.8888888888888857 0.4993185036986164

0.4473075704521675 0.8942882711521611 0.4998022745545507

0.7803140340333292 0.8942882711521611 0.4998022745545507

0.2246537287574561 0.1159741241815835 0.2458090124776199

0.5560667312780162 0.1113666989723438 0.2509265424630170

0.8886333010276563 0.1113666989723438 0.2509265424630170

0.2228328984765273 0.4447497825715959 0.2521597016770747

0.5552502174284047 0.4447497825715959 0.2521597016770747

0.8886333010276563 0.4439332687219840 0.2509265424630170

0.2246537287574561	0.7753462712425441	0.2458090124776199
0.5552502174284047	0.7771671015234726	0.2521597016770747
0.8840258758184170	0.7753462712425441	0.2458090124776199
0.2219234105573211	0.1105134877813138	0.7511780485632955
0.5560195529063980	0.1113431097865347	0.7520162543129676
0.8886568902134649	0.1113431097865347	0.7520162543129676
0.2174366109048124	0.4420516387857383	0.7460066881517818
0.5579483612142616	0.4420516387857383	0.7460066881517818
0.8886568902134649	0.4439804470936020	0.7520162543129676
0.2219234105573211	0.7780765894426788	0.7511780485632955
0.5579483612142616	0.7825633890951877	0.7460066881517818
0.8894865122186861	0.7780765894426788	0.7511780485632955
0.2353842281708715	0.1374351230084143	0.0193280321690978
0.5553474112874406	0.1110070389770560	0.0011853010549979
0.8889929610229439	0.1110070389770560	0.0011853010549979
0.2222141354341149	0.4444404010503896	-0.0178441376800902
0.5555595989496102	0.4444404010503895	-0.0178441376800902
0.8889929610229439	0.4446525887125594	0.0011853010549979
0.2353842281708715	0.7646157718291287	0.0193280321690978
0.5555595989496102	0.7777858645658852	-0.0178441376800902
0.8625648769915862	0.7646157718291287	0.0193280321690978
0.2223049046406581	0.1112764759479875	0.5009793459720316
0.5555558606653134	0.1111112636659922	0.4832548405398722
0.8888887363340076	0.1111112636659922	0.4832548405398722
0.1962214069967109	0.4314440368316879	0.5191735082962673
0.5685559631683120	0.4314440368316878	0.5191735082962673
0.8888887363340076	0.4444441393346868	0.4832548405398722
0.2223049046406581	0.7776950953593420	0.5009793459720316
0.5685559631683121	0.8037785930032886	0.5191735082962673
0.8887235240520125	0.7776950953593420	0.5009793459720316
0.1112514977662947	0.2224121408380556	0.2508554935563608
0.4444939764050967	0.2224121408380556	0.2508554935563608
0.7777777777777786	0.2222222222222214	0.2504581488959697

0.1112514977662947 0.5555060235949036 0.2508554935563608

0.4444444444444429 0.5555555555555571 0.2518580834457385

0.7775878591619443 0.5555060235949036 0.2508554935563608

0.1111111111111143 0.8888888888888857 0.2293361564129868

0.4444939764050967 0.8887485022337056 0.2508554935563608

0.7775878591619443 0.8887485022337056 0.2508554935563608

0.1109754876674482 0.2220355249681848 0.7508024091864365

0.4443933706340722 0.2220355249681848 0.7508024091864365

0.7777777777777786 0.2222222222222214 0.7514251818043292

0.1109754876674482 0.5556066293659275 0.7508024091864365

0.4444444444444429 0.5555555555555571 0.7292424446453482

0.7779644750318152 0.5556066293659275 0.7508024091864365

0.1111111111111143 0.8888888888888857 0.7522640486104882

0.4443933706340722 0.8890245123325516 0.7508024091864365

0.7779644750318152 0.8890245123325516 0.7508024091864365

0.1111111111111143 0.8888888888888857 0.0686387219121325

0.4444444444444429 0.5555555555555571 0.5686510070009848

KPOINTS used for structural relaxation, e_{ij} (using LCALCEPS method), and C_{ij} calculations

Automatic mesh
0
Gamma
3 3 2
0 0 0

e_{ij}^{ion} (C/m^2):

	XX	YY	ZZ	XY	YZ	ZX
x	0.00000	0.00000	0.00000	-0.08001	0.00000	-0.04690
y	-0.08001	0.08001	0.00000	-0.00000	-0.04690	0.00000
z	-0.29514	-0.29514	-0.10493	0.00000	0.00000	0.00000

e_{ij}^{elc} (C/m^2):

	XX	YY	ZZ	XY	YZ	ZX
x	0.00000	-0.00000	0.00000	-0.06151	0.00000	0.01429
y	-0.06151	0.06151	0.00000	0.00000	0.01429	0.00000
z	0.01783	0.01783	-0.05787	0.00000	0.00000	0.00000

Total e_{ij} (C/m^2):

	XX	YY	ZZ	XY	YZ	ZX
x	0.00000	0.00000	0.00000	-0.14152	0.00000	-0.03261
y	-0.14152	0.14152	0.00000	0.00000	-0.03261	0.00000
z	-0.27731	-0.27731	-0.16280	0.00000	0.00000	0.00000

C_{ij} (kBar) [1 kBar = 10^8 N/m^2]

Direction	XX	YY	ZZ	XY	YZ	ZX
XX	7301.8607	1396.6981	258.3082	0.0000	-10.6519	0.0000
YY	1396.6981	7301.8607	258.3082	-0.0000	10.6519	0.0000
ZZ	258.3082	258.3082	446.1133	0.0000	0.0000	0.0000
XY	0.0000	0.0000	0.0000	2952.5813	0.0000	-10.6519
YZ	-10.6519	10.6519	0.0000	0.0000	81.3443	0.0000
ZX	0.0000	0.0000	0.0000	-10.6519	0.0000	81.3444

d_{ij} (pm/V):

	XX	YY	ZZ	XY	YZ	ZX
x	0.00000	0.00000	0.00000	-0.49401	0.00000	-4.07357
y	-0.24700	0.24700	0.00000	0.00000	-4.07357	0.00000
z	-0.21793	-0.21793	-3.39693	0.00000	0.00000	0.00000

Protruded $Ga_{0.0556}B_{0.9444}N$ Structure ($3 \times 3 \times 2$ supercell):

Optimized Structure:

FE-$Ga_{0.0556}B_{0.9444}N$

1.00000000000000

7.5865206351450398 0.0000000000000000 0.0000000000000000

-3.7932603175725199 6.5701195963704633 0.0000000000000000

0.0000000000000000 0.0000000000000000 13.5422511568258113

B N Ga

34 36 2

Direct

0.1138268094109832 0.2287904007816330 -0.0020927868663686

0.4482969247039855 0.2287904007816330 -0.0020927868663686

0.7777777777777786 0.2222222222222214 -0.0019397574153558

0.1138268094109832 0.5517030752960144 -0.0020927868663686

0.4444444444444429 0.5555555555555571 -0.0224912548512381

0.7712095992183672 0.5517030752960144 -0.0020927868663686

0.4482969247039855 0.8861731905890167 -0.0020927868663686

0.7712095992183672 0.8861731905890167 -0.0020927868663686

0.1045177104299669 0.2184339528179581 0.4981640313958593

0.4472495757213268 0.2184339528179581 0.4981640313958593

0.7777777777777786 0.2222222222222214 0.4781661353793200

0.1045177104299669 0.5527504242786732 0.4981640313958593

0.7815660471820416 0.5527504242786732 0.4981640313958593

0.1111111111111143 0.8888888888888857 0.4982826017930019

0.4472495757213268 0.8954822895700331 0.4981640313958593

0.7815660471820416 0.8954822895700331 0.4981640313958593

0.2245421044429248 0.1157508755525209 0.2491853609167813

0.5560384447853814 0.1113525557260264 0.2517848408620033

0.8886474442739735 0.1113525557260264 0.2517848408620033

0.2227769458795330 0.4447218062730986 0.2524461534010496

0.5552781937269015 0.4447218062730986 0.2524461534010496

0.8886474442739735 0.4439615552146186 0.2517848408620033

0.2245421044429248	0.7754578955570752	0.2491853609167813
0.5552781937269013	0.7772230541204671	0.2524461534010496
0.8842491244474792	0.7754578955570752	0.2491853609167813
0.2219645838098969	0.1105958342864651	0.7519580031415734
0.5560002590505098	0.1113334628585906	0.7524313361053021
0.8886665371414095	0.1113334628585906	0.7524313361053021
0.2176563495253196	0.4421615080959921	0.7495114465942502
0.5578384919040085	0.4421615080959921	0.7495114465942502
0.8886665371414095	0.4439997409494904	0.7524313361053021
0.2219645838098969	0.7780354161901033	0.7519580031415734
0.5578384919040085	0.7823436504746801	0.7495114465942502
0.8894041657135349	0.7780354161901033	0.7519580031415734
0.2390946571510575	0.1448559809687865	0.0200437385370777
0.5553299733166617	0.1109983199916664	0.0002652198212963
0.8890016800083336	0.1109983199916664	0.0002652198212963
0.2218992955094876	0.4442829810880760	-0.0230018455874128
0.5557170189119239	0.4442829810880760	-0.0230018455874128
0.8890016800083336	0.4446700266833387	0.0002652198212963
0.2390946571510575	0.7609053428489426	0.0200437385370777
0.5557170189119239	0.7781007044905124	-0.0230018455874128
0.8551440190312133	0.7609053428489426	0.0200437385370777
0.2223139847280237	0.1112946361227190	0.5001901754449978
0.5552610447673356	0.1109638557170035	0.4779487590385844
0.8890361442829965	0.1109638557170035	0.4779487590385844
0.1888796020978587	0.4277731343822613	0.5199022334931860
0.5722268656177384	0.4277731343822613	0.5199022334931860
0.8890361442829965	0.4447389552326644	0.4779487590385844
0.2223139847280237	0.7776860152719766	0.5001901754449978
0.5722268656177384	0.8111203979021416	0.5199022334931860
0.8887053638772806	0.7776860152719766	0.5001901754449978
0.1112806539015709	0.2225222599084795	0.2517977003057943
0.4445749393402443	0.2225222599084795	0.2517977003057943
0.7777777777777786	0.2222222222222214	0.2505084273963463

0.1112806539015709 0.5554250606597556 0.2517977003057943

0.4444444444444429 0.5555555555555571 0.2520916389698538

0.7774777400915207 0.5554250606597556 0.2517977003057943

0.1111111111111143 0.8888888888888857 0.2373531097195161

0.4445749393402443 0.8887193460984291 0.2517977003057943

0.7774777400915207 0.8887193460984291 0.2517977003057943

0.1108630132675292 0.2219266827930078 0.7518052140632734

0.4443970028588143 0.2219266827930078 0.7518052140632734

0.7777777777777786 0.2222222222222214 0.7517693132280909

0.1108630132675292 0.5556029971411859 0.7518052140632734

0.4444444444444429 0.5555555555555571 0.7371272236841396

0.7780733172069919 0.5556029971411859 0.7518052140632734

0.1111111111111143 0.8888888888888857 0.7524993371174645

0.4443970028588143 0.8891369867324707 0.7518052140632734

0.7780733172069919 0.8891369867324707 0.7518052140632734

0.1111111111111143 0.8888888888888857 0.0728458697919186

0.4444444444444429 0.5555555555555571 0.5730465458717141

KPOINTS used for structural relaxation, e_{ij} (using LCALCEPS method), and C_{ij} calculations

Automatic mesh

0

Gamma

3 3 2

0 0 0

e_{ij}^{ion} (C/m^2):

	XX	YY	ZZ	XY	YZ	ZX
x	0.00000	-0.00000	0.00000	-0.05951	0.00000	-0.05857
y	-0.05951	0.05951	0.00000	-0.00000	-0.05857	0.00000
z	-0.32494	-0.32494	-0.09856	-0.00000	-0.00000	-0.00000

e_{ij}^{elc} (C/m^2):

	XX	YY	ZZ	XY	YZ	ZX
x	-0.00000	-0.00000	-0.00000	-0.04237	-0.00000	0.03255
y	-0.04237	0.04237	0.00000	0.00000	0.03255	0.00000
z	0.04448	0.04448	-0.10134	0.00000	0.00000	0.00000

Total e_{ij} (C/m^2):

	XX	YY	ZZ	XY	YZ	ZX
x	0.00000	0.00000	0.00000	-0.10188	0.00000	-0.02602
y	-0.10188	0.10188	0.00000	0.00000	-0.02602	0.00000
z	-0.28046	-0.28046	-0.19990	0.00000	0.00000	0.00000

C_{ij} (kBar) [1 kBar = 10^8 N/m^2]

Direction	XX	YY	ZZ	XY	YZ	ZX
XX	6912.5401	1220.7691	268.3449	0.0000	-12.5679	-0.0000
YY	1220.7691	6912.5401	268.3449	0.0000	12.5679	0.0000
ZZ	268.3449	268.3449	410.8453	0.0000	-0.0000	-0.0000
XY	0.0000	0.0000	0.0000	2845.8855	-0.0000	-12.5679
YZ	-12.5679	12.5679	-0.0000	-0.0000	73.3993	-0.0000
ZX	-0.0000	0.0000	-0.0000	-12.5679	-0.0000	73.3993

d_{ij} (pm/V):

	XX	YY	ZZ	XY	YZ	ZX
x	0.00000	0.00000	0.00000	-0.37393	0.00000	-3.60902
y	-0.18696	0.18696	0.00000	0.00000	-3.60902	0.00000
z	-0.19260	-0.19260	-4.61399	0.00000	0.00000	0.00000

Protruded $Al_{0.1250}B_{0.8750}N$ Structure ($2 \times 2 \times 4$ supercell):

Optimized Structure:

FE-$Al_{0.1250}B_{0.8750}N$

1.00000000000000

5.1360312144188658 0.0000000000000000 0.0000000000000000

-2.5680156072094329 4.4482010455380170 -0.0080356599044738

0.0000000000000000 -0.0447993454245307 24.7467433676581017

B N Al

28 32 4

Direct

0.1714525450688298 0.3295680518842314 0.0026308249125290

0.6697001711398736 0.3239077668211663 0.0021277252621784

0.1777227407294064 0.8323583304202308 0.0016182842328081

0.6693151590487244 0.8331213842079388 0.0016377847702293

0.1638057341092049 0.3331213841603679 0.2516377847693553

0.6546350986894657 0.3323583304643696 0.2516182842319342

0.1581150008135693 0.8295680518796911 0.2526308249117261

0.6542071197278437 0.8239077668222964 0.2521277252612548

0.1697001561398760 0.3239077668211663 0.5021277252621783

0.6714525600688274 0.3295680518842314 0.5026308249125290

0.1693151440487266 0.8331213842079388 0.5016377847702294

0.6777227557294040 0.8323583304202308 0.5016182842328152

0.1546350836894679 0.3323583304643696 0.7516182842319341

0.1542071047278459 0.8239077668222964 0.7521277252612546

0.3497060195291127 0.1773966748985644 0.1270507521648312

0.8413312647174424 0.6679113403485131 0.1243179977487935

0.3496953689476042 0.6666998105590459 0.1271118829591174

0.8265785256311771 0.1679113253173865 0.3743179977473795

0.3170029516398326 0.1666997955842938 0.3771118829577034

0.8276891503424723 0.6773966898954784 0.3770507521635948

0.8496953389476016 0.6666998105590459 0.6271118829591176

0.3413312947174449 0.6679113403485131 0.6243179977487935

0.8170029216398299	0.1666997955842938	0.8771118829576965
0.3265785556311794	0.1679113253173865	0.8743179977473795
0.3276891803424747	0.6773966898954784	0.8770507521635948
0.6638057491092025	0.3331213841603679	0.7516377847693554
0.8497059895291103	0.1773966748985644	0.6270507521648312
0.6581150158135671	0.8295680518796911	0.7526308249117261
0.3386649325632915	0.1628286518589876	0.0024975324559123
0.8388385458649958	0.1632679396885301	0.0027369094133572
0.3383786552833200	0.6629933526219376	0.0029404024815738
0.8388576398219308	0.6625850024827847	0.9937450177848730
0.3246151733399006	0.1629933376198937	0.2529404024807709
0.8237278386587690	0.1625849874802434	0.2437450177838570
0.3244298848222570	0.6632679546894018	0.2527369094124548
0.8241642102913209	0.6628286668539054	0.2524975324550738
0.3388385458649957	0.1632679396885301	0.5027369094133571
0.8386649325632916	0.1628286518589876	0.5024975324559122
0.3388576398219307	0.6625850024827847	0.4937450177848731
0.8383786552833199	0.6629933526219376	0.5029404024815739
0.3237278386587689	0.1625849874802434	0.7437450177838569
0.8246151733399005	0.1629933376198937	0.7529404024807710
0.3241642102913206	0.6628286668539054	0.7524975324550738
0.8244298848222568	0.6632679546894018	0.7527369094124547
0.2074802047799311	0.3509985412288927	0.1348288873391559
0.6696449565401872	0.3483276301600834	0.1224386140047391
0.1806277430756781	0.8375280136431961	0.1273792869111577
0.6695382982797194	0.8155022194455014	0.1227155816414146
0.1459634301851141	0.3155022194426520	0.3727155816400431
0.6568997795665003	0.3375280136402474	0.3773792869097651
0.1435178304471079	0.8509985412452422	0.3848288873370172
0.6786821975962453	0.8483276301354703	0.3724386140030905
0.1696449415401897	0.3483276301600834	0.6224386140047389
0.7074802197799287	0.3509985412288927	0.6348288873391560
0.1695382832797219	0.8155022194455014	0.6227155816414147

0.6806277580756757 0.8375280136431961 0.6273792869111579

0.1568997645665027 0.3375280136402474 0.8773792869097652

0.6459634451851117 0.3155022194426520 0.8727155816400434

0.1786821825962477 0.8483276301354703 0.8724386140030904

0.6435178454471054 0.8509985412452422 0.8848288873370173

0.8342916527949514 0.1650052297493192 0.1542225159257963

0.3307120719542185 0.6650052447501340 0.4042225159265211

0.3342916827949538 0.1650052297493192 0.6542225159257964

0.8307120419542161 0.6650052447501340 0.9042225159265211

KPOINTS used for structural relaxation, e_{ij} (using LCALCEPS method), and C_{ij} calculations

Automatic mesh

0

Gamma

6 6 3

0 0 0

e_{ij}^{ion} (C/m^2):

	XX	YY	ZZ	XY	YZ	ZX
x	-0.00000	0.00000	-0.00000	0.00871	0.00000	0.06407
y	-0.19619	0.39412	-0.02214	0.00000	0.26585	0.00000
z	-0.39586	-0.47545	-0.66265	0.00000	-0.01459	0.00000

e_{ij}^{elc} (C/m^2):

	XX	YY	ZZ	XY	YZ	ZX
x	0.00000	0.00000	0.00000	0.14864	0.00000	0.08988
y	0.14844	-0.14654	0.00663	0.00000	0.07279	0.00000
z	0.06711	0.05687	-0.24075	0.00000	-0.00577	0.00000

Total e_{ij} (C/m^2):

	XX	YY	ZZ	XY	YZ	ZX
x	0.00000	0.00000	0.00000	0.15735	0.00000	0.15395
y	-0.04775	0.24758	-0.01551	0.00000	0.33864	0.00000
z	-0.32875	-0.41858	-0.90340	0.00000	-0.02036	0.00000

C_{ij} (kBar) [1 kBar = 10^8 N/m^2]

Direction	XX	YY	ZZ	XY	YZ	ZX
XX	6049.8068	833.4718	145.3872	-0.0000	105.5461	0.0000
YY	833.4718	6465.0996	135.8037	-0.0000	-108.6353	0.0000
ZZ	145.3872	135.8037	553.4770	-0.0000	4.4925	-0.0000
XY	-0.0000	0.0000	0.0000	2751.4518	0.0000	54.6585
YZ	105.5461	-108.6353	4.4925	0.0000	97.0809	-0.0000
ZX	0.0000	0.0000	-0.0000	54.6585	-0.0000	120.3989

d_{ij} (pm/V):

	XX	YY	ZZ	XY	YZ	ZX
x	0.00000	0.00000	0.00000	0.32076	0.00000	12.64104
y	-0.86730	1.13178	-0.63141	0.00000	37.12087	0.00000
z	-0.08100	-0.32378	-16.20839	0.00000	-1.62142	0.00000

Protruded $Ga_{0.1250}B_{0.8750}N$ Structure ($2 \times 2 \times 4$ supercell):

Optimized Structure:

FE-$Ga_{0.1250}B_{0.8750}N$

1.00000000000000

5.1322933759636920 0.0000000000000000 0.0000000000000000

-2.5661466879818460 4.4459006401445844 -0.0247267662947655

0.0000000000000000 -0.1316425412395223 25.3355799489459699

B N Ga

28 32 4

Direct

0.1787475712095352 0.3257803624897353 0.0035957036856446

0.6777427997364485 0.3207912587673741 0.0040581122373826

0.1845553081117124 0.8281571628334770 0.0031420804907190

0.6771532897164445 0.8293888593491356 0.0040106720174432

0.1522350786326897 0.3293888593491358 0.2540106720174428

0.6436013637217629 0.3281571628334770 0.2531420804907188

0.1470322852802008 0.8257803624897351 0.2535957036856445

0.6430479830309219 0.8207912587673745 0.2540581122373828

0.1777427847364510 0.3207912587673741 0.5040581122373825

0.6787475862095330 0.3257803624897353 0.5035957036856450

0.1771532747164468 0.8293888593491356 0.5040106720174437

0.6845553231117094 0.8281571628334770 0.5031420804907192

0.1436013487217655 0.3281571628334770 0.7531420804907192

0.1430479680309241 0.8207912587673745 0.7540581122373825

0.3637725237905922 0.1808510414368139 0.1259966303781135

0.8553720584664827 0.6712423903560600 0.1207265436405037

0.3638378605209267 0.6705036464527487 0.1261728817002166

0.8158687818895769 0.1712423753560622 0.3707265436405035

0.3066642959318266	0.1705036314527512	0.3761728817002166
0.8170770126462220	0.6808510564368121	0.3759966303781133
0.8638378305209240	0.6705036464527487	0.6261728817002163
0.3553720884664850	0.6712423903560599	0.6207265436405037
0.8066642659318241	0.1705036314527512	0.8761728817002163
0.3158688118895793	0.1712423753560622	0.8707265436405037
0.3170770426462241	0.6808510564368121	0.8759966303781136
0.6522350936326878	0.3293888593491358	0.7540106720174437
0.8637724937905902	0.1808510414368139	0.6259966303781136
0.6470323002801990	0.8257803624897351	0.7535957036856450
0.3459360386301135	0.1588851906706575	0.0031334955561378
0.8465911647907658	0.1596583956113487	0.0037812683856506
0.3455570185975239	0.6590176957758903	0.0042226900271487
0.8461429274295625	0.6587123059223281	-0.0029443120540416
0.3134611531783699	0.1590176807758924	0.2542226900271489
0.8125698544927691	0.1587122909223305	0.2470556879459583
0.3130677218205850	0.6596584106113460	0.2537812683856508
0.8129496430405458	0.6588852056706549	0.2531334955561381
0.3465911647907653	0.1596583956113487	0.5037812683856503
0.8459360386301138	0.1588851906706575	0.5031334955561375
0.3461429274295623	0.6587123059223281	0.4970556879459584
0.8455570185975245	0.6590176957758903	0.5042226900271488
0.3125698544927697	0.1587122909223305	0.7470556879459587
0.8134611531783696	0.1590176807758924	0.7542226900271488
0.3129496430405455	0.6588852056706549	0.7531334955561375
0.8130677218205845	0.6596584106113460	0.7537812683856504
0.2357224970501880	0.3621666212810359	0.1394782416726847
0.6813540224702092	0.3519351847985566	0.1162229044594345
0.1934381537522244	0.8404485970264453	0.1258981474508499

0.6814737441988158 0.8161176513204293 0.1169086920293405

0.1346434161216121 0.3161176513204293 0.3669086920293406

0.6470099522742185 0.3404485970264447 0.3758981474508500

0.1264436182308487 0.8621666212810362 0.3894782416726845

0.6705806863283432 0.8519351847985558 0.3662229044594345

0.1813540074702120 0.3519351847985566 0.6162229044594344

0.7357225120501854 0.3621666212810359 0.6394782416726842

0.1814737291988181 0.8161176513204293 0.6169086920293398

0.6934381687522226 0.8404485970264453 0.6258981474508498

0.1470099372742209 0.3404485970264447 0.8758981474508498

0.6346434311216098 0.3161176513204293 0.8669086920293398

0.1705806713283453 0.8519351847985558 0.8662229044594344

0.6264436332308462 0.8621666212810362 0.8894782416726842

0.8407482530903952 0.1663435759079734 0.1555962483227689

0.3255938178175776 0.6663435909079710 0.4055962483227689

0.3407482830903978 0.1663435759079734 0.6555962483227687

0.8255937878175751 0.6663435909079710 0.9055962483227687

KPOINTS used for structural relaxation, e_{ij} (using LCALCEPS method), and C_{ij} calculations

Automatic mesh
0
Gamma
6 6 3
0 0 0

e_{ij}^{ion} (C/m^2):

	XX	YY	ZZ	XY	YZ	ZX
x	0.00000	0.00000	0.00000	0.03599	0.00000	-0.09359
y	-0.22641	0.24844	-0.01439	0.00000	0.09480	0.00000
z	-0.56063	-0.65128	-0.80810	0.00000	-0.02685	-0.00000

e_{ij}^{elc} (C/m^2):

	XX	YY	ZZ	XY	YZ	ZX
x	0.00000	0.00000	0.00000	0.08234	0.00000	0.15098
y	0.08923	-0.12459	0.03729	0.00000	0.12353	0.00000
z	0.12727	0.12392	-0.39788	0.00000	-0.00114	0.00000

Total e_{ij} (C/m^2):

	XX	YY	ZZ	XY	YZ	ZX
x	0.00000	0.00000	0.00000	0.11833	0.00000	0.05739
y	-0.13718	0.12385	0.02290	0.00000	0.21833	0.00000
z	-0.43336	-0.52736	-1.20598	0.00000	-0.02799	0.00000

C_{ij} (kBar) [1 kBar = 10^8 N/m^2]

Direction	XX	YY	ZZ	XY	YZ	ZX
XX	5826.8288	848.8388	152.4272	0.0000	111.3514	0.0000
YY	848.8388	6105.6880	155.6417	-0.0000	-170.4310	-0.0000
ZZ	152.4272	155.6417	453.1155	-0.0000	3.0605	-0.0000
XY	0.0000	-0.0000	-0.0000	2641.0715	-0.0000	56.7814
YZ	111.3514	-170.4310	3.0605	-0.0000	106.2563	0.0000
ZX	0.0000	-0.0000	-0.0000	56.7814	0.0000	126.8166

d_{ij} (pm/V):

	XX	YY	ZZ	XY	YZ	ZX
x	0.00000	0.00000	0.00000	0.35415	0.00000	4.36686
y	-0.81951	0.94889	0.30033	0.00000	22.91962	0.00000
z	0.03171	-0.25667	-26.52215	0.00000	-2.31520	0.00000

Protruded $Al_{0.0625}B_{0.9375}N$ Structure ($2 \times 2 \times 2$ supercell):

FE-$Al_{0.0625}B_{0.9375}N$

1.00000000000000

5.0799955394631651 -0.0000000000000005 0.0000000000000000

-2.5399977697315825 4.3994051882867335 -0.0000000000000000

0.0000000000000000 0.0000000000000000 12.9682112483147005

B N Al

15 16 1

Direct

x	y	z	Z_{33}	$\frac{du_3}{d\eta_3}$
0.1666963425562735	0.3333036574437264	0.9847904143497390	0.90398	-0.02522
0.6666073148874742	0.3333036574437264	0.9847904143497390	0.90398	-0.02522
0.1666963425562735	0.8333926851125258	0.9847904143497390	0.90398	-0.02522
0.6666666666666714	0.8333333333333286	0.9850524254722184	0.82220	-0.02654
0.1642251021667667	0.3357748978332332	0.4890406804794243	0.52386	0.07178
0.6715497956664734	0.3357748978332332	0.4890406804794243	0.52386	0.07178
0.1642251021667667	0.8284502043335266	0.4890406804794243	0.52386	0.07178
0.3333333333333286	0.1666666666666714	0.2408801574879142	0.84168	0.02406
0.8331587165978241	0.1663174331956691	0.2410356285681091	0.87570	0.02104
0.3336825668043311	0.6668412834021759	0.2410356285681091	0.87570	0.02104
0.8331587165978241	0.6668412834021759	0.2410356285681091	0.87570	0.02104
0.3333333333333286	0.1666666666666714	0.7301454418073831	1.03542	-0.06324
0.8359583152141994	0.1719166304284125	0.7278323577570427	1.08517	-0.06919
0.3280833695715871	0.6640416847858006	0.7278323577570427	1.08517	-0.06919
0.8359583152141994	0.6640416847858006	0.7278323577570427	1.08517	-0.06919
0.3333333333333286	0.1666666666666714	0.9839194081866292	-0.88363	-0.02177
0.8333078605424773	0.1666157210849769	0.9835839861995068	-0.87691	-0.01833
0.3333842789150233	0.6666921394575227	0.9835839861995068	-0.87691	-0.01833

0.8333078605424773 0.6666921394575227 0.9835839861995068 -0.87691 -0.01833

0.3333333333333286 0.1666666666666714 0.4944954480188792 -0.82656 0.06104

0.8447153722602770 0.1894307445205614 0.4907548160271712 -0.92356 0.07243

0.3105692554794386 0.6552846277397230 0.4907548160271712 -0.92356 0.07243

0.8447153722602770 0.6552846277397230 0.4907548160271712 -0.92356 0.07243

0.1666805586973344 0.3333194413026657 0.2399833439964360 -0.89161 0.02812

0.6666388826053455 0.3333194413026657 0.2399833439964360 -0.89161 0.02812

0.1666805586973344 0.8333611173946545 0.2399833439964360 -0.89161 0.02812

0.6666666666666714 0.8333333333333286 0.2392026482625952 -0.83725 0.02879

0.1666209300756898 0.3333790699243102 0.7322700373683941 -0.85653 -0.06836

0.6667581398486346 0.3333790699243102 0.7322700373683941 -0.85653 -0.06836

0.1666209300756898 0.8332418601513654 0.7322700373683941 -0.85653 -0.06836

0.6666666666666714 0.8333333333333286 0.7157804718358300 -1.25693 -0.05325

0.6666666666666714 0.8333333333333286 0.5426502046910957 1.57965 0.01406

e_{ij}^{ion} (C/m^2) using the LCALCEPS = .True. and k-point mesh of $3 \times 3 \times 3$:

	XX	YY	ZZ	XY	YZ	ZX
x	-0.00000	-0.00000	0.00000	-0.12360	0.00000	-0.04922
y	-0.12360	0.12360	0.00000	0.00000	-0.04922	-0.00000
z	-0.22999	-0.22999	-0.15187	-0.00000	0.00000	-0.00000

e_{ij}^{elc} (C/m^2) using the LCALCEPS = .True. and k-point mesh of $3 \times 3 \times 3$:

	XX	YY	ZZ	XY	YZ	ZX
x	-0.00000	0.00000	0.00000	-0.07535	-0.00000	0.02885
y	-0.07535	0.07535	-0.00000	0.00000	0.02885	-0.00000
z	0.01357	0.01357	-0.07581	-0.00000	0.00000	-0.00000

C_{ij} (kBar) [1 kBar = 10^8 N/m^2] using IBRION = 6 and k-point mesh of $6 \times 6 \times 6$:

Direction	XX	YY	ZZ	XY	YZ	ZX
XX	7873.1713	1700.2602	23.3171	-0.0000	-3.9898	0.0000
YY	1700.2602	7873.1713	23.3171	-0.0000	3.9898	-0.0000
ZZ	23.3171	23.3171	439.7964	0.0000	-0.0000	0.0000
XY	0.0000	-0.0000	-0.0000	3086.4555	0.0000	-3.9898
YZ	-3.9898	3.9898	0.0000	0.0000	78.4502	-0.0000
ZX	0.0000	-0.0000	0.0000	-3.9898	-0.0000	78.4502

d_{ij} (pm/V):

	XX	YY	ZZ	XY	YZ	ZX
x	0.00000	0.00000	0.00000	-0.64799	0	-2.62951
y	-0.32399	0.32399	0.00000	0	-2.62951	0.00000
z	-0.21351	-0.21351	-5.15430	0	-0.00000	0.00000

Convergence of e_{ij}^{ion}, e_{ij}^{elc}, C_{ij}, and d_{ij} in terms of k-point mesh (cutoff energy for the plane-wave-basis set, ENCUT = 500 eV):

e_{ij}^{ion} (C/m^2) using DFPT and k-point mesh of $3 \times 3 \times 3$:

	XX	YY	ZZ	XY	YZ	ZX
x	0.00008	-0.00008	-0.00000	-0.12508	-0.00001	-0.04884
y	-0.12510	0.12511	-0.00000	0.00000	-0.04868	0.00000
z	-0.24194	-0.24194	-0.16266	-0.00000	-0.00000	-0.00000

e_{ij}^{elc} (C/m^2) using DFPT and k-point mesh of $3 \times 3 \times 3$:

	XX	YY	ZZ	XY	YZ	ZX
x	-0.00000	0.00000	0.00000	-0.07643	-0.00000	0.02753
y	-0.07643	0.07643	0.00000	0.00000	0.02753	-0.00000
z	0.01419	0.01419	-0.07747	-0.00000	-0.00000	0.00000

e_{ij}^{ion} (C/m^2) using DFPT and k-point mesh of $6 \times 6 \times 6$:

	XX	YY	ZZ	XY	YZ	ZX
x	0.00006	-0.00006	0.00000	-0.12362	-0.00000	-0.04807
y	-0.12363	0.12362	-0.00000	0.00000	-0.04791	-0.00000
z	-0.23792	-0.23792	-0.15791	0.00000	-0.00000	0.00000

e_{ij}^{elc} (C/m^2) using DFPT and k-point mesh of $6 \times 6 \times 6$:

	XX	YY	ZZ	XY	YZ	ZX
x	-0.00000	0.00000	0.00000	-0.07511	-0.00000	0.02785
y	-0.07511	0.07511	-0.00000	0.00000	0.02785	-0.00000
z	0.01428	0.01428	-0.07659	-0.00000	0.00000	-0.00000

e_{ij}^{ion} (C/m^2) using DFPT and k-point mesh of $9 \times 9 \times 9$:

	XX	YY	ZZ	XY	YZ	ZX
x	0.00002	-0.00002	0.00000	-0.12361	-0.00000	-0.04808
y	-0.12361	0.12361	0.00000	-0.00000	-0.04788	-0.00000
z	-0.24214	-0.24214	-0.16455	-0.00000	-0.00000	-0.00000

e^{elc}_{ij} (C/m^2) using DFPT and k-point mesh of $9 \times 9 \times 9$:

	XX	YY	ZZ	XY	YZ	ZX
x	-0.00000	0.00000	0.00000	-0.07512	-0.00000	0.02787
y	-0.07512	0.07512	0.00000	0.00000	0.02787	-0.00000
z	0.01431	0.01431	-0.07658	-0.00000	0.00000	0.00000

C_{ij} (kBar) [1 kBar = 10^8 N/m^2] using IBRION = 6 and k-point mesh of $3 \times 3 \times 3$:

Direction	XX	YY	ZZ	XY	YZ	ZX
XX	7872.0544	1700.8357	23.7179	0.0000	-3.9407	-0.0000
YY	1700.8357	7872.0544	23.7179	-0.0000	3.9407	0.0000
ZZ	23.7179	23.7179	440.8163	-0.0000	0.0000	0.0000
XY	-0.0000	-0.0000	0.0000	3085.6093	-0.0000	-3.9407
YZ	-3.9407	3.9407	0.0000	-0.0000	78.9511	-0.0000
ZX	-0.0000	0.0000	0.0000	-3.9407	-0.0000	78.9511

C_{ij} (kBar) [1 kBar = 10^8 N/m^2] using IBRION = 6 and k-point mesh of $6 \times 6 \times 6$:

Direction	XX	YY	ZZ	XY	YZ	ZX
XX	7873.1713	1700.2602	23.3171	-0.0000	-3.9898	0.0000
YY	1700.2602	7873.1713	23.3171	-0.0000	3.9898	-0.0000
ZZ	23.3171	23.3171	439.7964	0.0000	-0.0000	0.0000
XY	0.0000	-0.0000	-0.0000	3086.4555	0.0000	-3.9898
YZ	-3.9898	3.9898	0.0000	0.0000	78.4502	-0.0000
ZX	0.0000	-0.0000	0.0000	-3.9898	-0.0000	78.4502

C_{ij} (kBar) [1 kBar = 10^8 N/m^2] using IBRION = 6 and k-point mesh of $9 \times 9 \times 9$:

Direction	XX	YY	ZZ	XY	YZ	ZX
XX	7873.0275	1699.8825	22.8968	-0.0000	-4.0450	-0.0000
YY	1699.8825	7873.0275	22.8968	-0.0000	4.0450	0.0000
ZZ	22.8968	22.8968	439.2665	0.0000	0.0000	0.0000

XY -0.0000 -0.0000 0.0000 3086.5725 0.0000 -4.0450

YZ -4.0450 4.0450 0.0000 0.0000 78.4782 0.0000

ZX -0.0000 0.0000 0.0000 -4.0450 0.0000 78.4782

----

d_{ij} (pm/V) using e_{ij} (C/m^2) using DFPT and C_{ij} using IBRION = 6 and k-point mesh of $3 \times 3 \times 3$:

	XX	YY	ZZ	XY	YZ	ZX
x	0.00013	-0.00013	0.00000	-0.65655	-0.00125	-2.73191
y	-0.32830	0.32831	0.00000	0.00000	-2.71165	0.00000
z	-0.22447	-0.22447	-5.42324	0.00000	0.00000	0.00000

d_{ij} (pm/V) using e_{ij} (C/m^2) using DFPT and C_{ij} using IBRION = 6 and k-point mesh of $6 \times 6 \times 6$:

	XX	YY	ZZ	XY	YZ	ZX
x	0.00010	-0.00010	0.00000	-0.64725	0.00001	-2.61035
y	-0.32363	0.32362	0.00000	0.00000	-2.58995	0.00000
z	-0.22068	-0.22068	-5.30861	0.00000	0.00000	0.00000

d_{ij} (pm/V) using e_{ij} (C/m^2) using DFPT and C_{ij} using IBRION = 6 and k-point mesh of $9 \times 9 \times 9$:

	XX	YY	ZZ	XY	YZ	ZX
x	0.00003	-0.00003	0.00000	-0.64727	0.00000	-2.60860
y	-0.32362	0.32362	0.00000	0.00000	-2.58311	0.00000
z	-0.22492	-0.22492	-5.46593	0.00000	0.00000	0.00000

Convergence in terms of the cutoff energy for the plane-wave-basis set (ENCUT):

e_{ij}^{ion} (C/m^2) using DFPT, k-point mesh of $6 \times 6 \times 6$, and ENCUT=500 eV:

	XX	YY	ZZ	XY	YZ	ZX
x	0.00006	-0.00006	0.00000	-0.12362	-0.00000	-0.04807
y	-0.12363	0.12362	-0.00000	0.00000	-0.04791	-0.00000
z	-0.23792	-0.23792	-0.15791	0.00000	-0.00000	0.00000

e_{ij}^{elc} (C/m^2) using DFPT, k-point mesh of $6 \times 6 \times 6$, and ENCUT=500 eV:

	XX	YY	ZZ	XY	YZ	ZX
x	-0.00000	0.00000	0.00000	-0.07511	-0.00000	0.02785
y	-0.07511	0.07511	-0.00000	0.00000	0.02785	-0.00000
z	0.01428	0.01428	-0.07659	-0.00000	0.00000	-0.00000

C_{ij} (kBar) [1 kBar = 10^8 N/m^2] using IBRION = 6, k-point mesh of $6 \times 6 \times 6$, and ENCUT=500 eV:

Direction	XX	YY	ZZ	XY	YZ	ZX
XX	7873.1713	1700.2602	23.3171	-0.0000	-3.9898	0.0000
YY	1700.2602	7873.1713	23.3171	-0.0000	3.9898	-0.0000
ZZ	23.3171	23.3171	439.7964	0.0000	-0.0000	0.0000
XY	0.0000	-0.0000	-0.0000	3086.4555	0.0000	-3.9898
YZ	-3.9898	3.9898	0.0000	0.0000	78.4502	-0.0000
ZX	0.0000	-0.0000	0.0000	-3.9898	-0.0000	78.4502

d_{ij} (pm/V) from e_{ij} (C/m^2) using DFPT and C_{ij} using IBRION = 6, k-point mesh of $6 \times 6 \times 6$, and ENCUT=500 eV:

	XX	YY	ZZ	XY	YZ	ZX
x	0.00010	-0.00010	0.00000	-0.64725	0.00001	-2.61035
y	-0.32363	0.32362	0.00000	0.00000	-2.58995	0.00000
z	-0.22068	-0.22068	-5.30861	0.00000	0.00000	0.00000

e_{ij}^{ion} (C/m^2) using DFPT, k-point mesh of $6 \times 6 \times 6$, and ENCUT=550 eV:

	XX	YY	ZZ	XY	YZ	ZX
x	0.00006	-0.00006	0.00000	-0.12388	-0.00000	-0.04804
y	-0.12389	0.12389	-0.00000	0.00000	-0.04788	0.00000
z	-0.23838	-0.23838	-0.15858	-0.00000	-0.00000	-0.00000

e_{ij}^{elc} (C/m^2) using DFPT, k-point mesh of $6 \times 6 \times 6$, and ENCUT=550 eV:

	XX	YY	ZZ	XY	YZ	ZX
x	-0.00000	0.00000	0.00000	-0.07512	-0.00000	0.02789
y	-0.07512	0.07512	-0.00000	0.00000	0.02789	-0.00000
z	0.01426	0.01426	-0.07648	-0.00000	-0.00000	0.00000

C_{ij} (kBar) [1 kBar = 10^8 N/m^2] using IBRION = 6, k-point mesh of $6 \times 6 \times 6$, and ENCUT=550 eV:

Direction	XX	YY	ZZ	XY	YZ	ZX
XX	7834.1041	1690.0236	7.5564	0.0000	-4.0961	0.0000
YY	1690.0236	7834.1041	7.5564	-0.0000	4.0961	0.0000
ZZ	7.5564	7.5564	380.2023	-0.0000	0.0000	-0.0000
XY	0.0000	-0.0000	-0.0000	3072.0403	-0.0000	-4.0961
YZ	-4.0961	4.0961	0.0000	-0.0000	60.6126	-0.0000
ZX	0.0000	0.0000	-0.0000	-4.0961	-0.0000	60.6127

d_{ij} (pm/V) from e_{ij} (C/m^2) using DFPT and C_{ij} using IBRION = 6, k-point mesh of $6 \times 6 \times 6$, and ENCUT=550 eV:

	XX	YY	ZZ	XY	YZ	ZX
x	0.00010	-0.00010	0.00000	-0.65227	0.00001	-3.36847
y	-0.32613	0.32613	0.00000	0.00000	-3.34207	0.00000
z	-0.23042	-0.23042	-6.17334	0.00000	0.00000	0.00000

e_{ij}^{ion} (C/m^2) using DFPT, k-point mesh of $6 \times 6 \times 6$, and ENCUT=600 eV:

	XX	YY	ZZ	XY	YZ	ZX
x	0.00006	-0.00006	0.00000	-0.12398	-0.00000	-0.04809
y	-0.12399	0.12399	-0.00000	0.00000	-0.04793	0.00000
z	-0.23854	-0.23854	-0.15845	0.00000	-0.00000	0.00000

e_{ij}^{elc} (C/m^2) using DFPT, k-point mesh of $6 \times 6 \times 6$, and ENCUT=600 eV:

	XX	YY	ZZ	XY	YZ	ZX
x	-0.00000	0.00000	0.00000	-0.07512	-0.00000	0.02790
y	-0.07512	0.07512	-0.00000	0.00000	0.02790	-0.00000
z	0.01427	0.01427	-0.07647	-0.00000	0.00000	-0.00000

C_{ij} (kBar) [1 kBar = 10^8 N/m^2] using IBRION = 6, k-point mesh of $6 \times 6 \times 6$, and ENCUT=600 eV:

Direction	XX	YY	ZZ	XY	YZ	ZX
XX	7845.3125	1695.0148	9.4294	0.0000	-4.0817	0.0000
YY	1695.0148	7845.3125	9.4294	-0.0000	4.0817	0.0000
ZZ	9.4294	9.4294	377.4757	-0.0000	-0.0000	-0.0000
XY	0.0000	0.0000	-0.0000	3075.1489	-0.0000	-4.0817
YZ	-4.0817	4.0817	0.0000	-0.0000	60.8911	0.0000
ZX	0.0000	-0.0000	-0.0000	-4.0817	0.0000	60.8910

d_{ij} (pm/V) from e_{ij} (C/m^2) using DFPT and C_{ij} using IBRION = 6, k-point mesh of $6 \times 6 \times 6$, and ENCUT=600 eV:

	XX	YY	ZZ	XY	YZ	ZX
x	0.00010	-0.00010	0.00000	-0.65191	0.00001	-3.35946
y	-0.32595	0.32595	0.00000	0.00000	-3.33318	0.00000
z	-0.22894	-0.22894	-6.21201	0.00000	0.00000	0.00000

Protruded $Al_{0.1250}B_{0.8750}N$ Structure ($2 \times 2 \times 2$ supercell):

FE-$Al_{0.1250}B_{0.8750}N$

1.00000000000000

5.1350966594585952 0.0004851038843495 -0.0178442468452302

-2.5671282174419763 4.4473667099219272 0.0178442468452302

-0.0429057337100513 0.0247716369072765 12.3656020191812352

B N Al

14 16 2

Direct

x	y	z	Z_{33}	$\frac{du_3}{d\eta_3}$
0.6669741212275263	0.3223713596294751	0.9853076291038461	0.61799	0.06938
0.1776286403705248	0.8330258787724737	0.9853076291038461	0.61799	0.06938
0.6680782120162915	0.8319217879837085	0.9796126059013537	0.44059	0.05176
0.1680782120162916	0.3319217879837085	0.4796126059013534	0.44059	0.05176
0.6776286403705249	0.3330258787724664	0.4853076291038459	0.61799	0.06938
0.1669741212275266	0.8223713596294751	0.4853076291038459	0.61799	0.06938
0.3324724249261158	0.1675275750738842	0.2336405261509062	1.52766	-0.06551
0.8237062307579223	0.1674459588604882	0.2356730663454876	1.47031	-0.07993
0.3325540411395119	0.6762937692420777	0.2356730663454876	1.47031	-0.07993
0.8293594066085069	0.6706405933914931	0.2366231369877130	1.20426	-0.05622
0.3293594066085069	0.1706405933914932	0.7366231369877132	1.20426	-0.05622
0.8325540411395119	0.1762937692420849	0.7356730663454873	1.47031	-0.07993
0.3237062307579150	0.6674459588604881	0.7356730663454873	1.47031	-0.07993
0.8324724249261158	0.6675275750738842	0.7336405261509061	1.52766	-0.06551
0.3514337552606578	0.1485662447393422	0.0013048345732121	-1.27729	0.11186
0.8159053193583993	0.1515050327695166	0.9759069371296694	-1.25849	0.05913
0.3484949672304833	0.6840946806416007	0.9759069371296694	-1.25849	0.05913
0.8377725921239535	0.6622274078760465	0.9860297901025004	-1.08383	0.05955

0.3377725921239536	0.1622274078760466	0.4860297901025002	-1.08383	0.05955
0.8484949672304835	0.1840946806416006	0.4759069371296692	-1.25849	0.05913
0.3159053193583994	0.6515050327695165	0.4759069371296692	-1.25849	0.05913
0.8514337552606578	0.6485662447393422	0.5013048345732122	-1.27729	0.11186
0.1623082492143806	0.3376917507856194	0.2188090244170510	-1.69300	-0.03537
0.6628866147331437	0.3372375261715402	0.2373161574590319	-1.10155	-0.04766
0.1627624738284599	0.8371133852668563	0.2373161574590319	-1.10155	-0.04766
0.6627658368166105	0.8372341631833895	0.2359650055175537	-1.02443	-0.05317
0.1627658368166103	0.3372341631833896	0.7359650055175537	-1.02443	-0.05317
0.6627624738284600	0.3371133852668565	0.7373161574590319	-1.10155	-0.04766
0.1628866147331437	0.8372375261715400	0.7373161574590319	-1.10155	-0.04766
0.6623082492143805	0.8376917507856195	0.7188090244170510	-1.69300	-0.03537
0.1648971143875117	0.3351028856124882	0.0396074962736667	2.46324	-0.01476
0.6648971143875119	0.8351028856124881	0.5396074962736666	2.46324	-0.01476

e_{ij}^{ion} (C/m^2) using the LCALCEPS = .True. and k-point mesh of $3 \times 3 \times 3$:

	XX	YY	ZZ	XY	YZ	ZX
x	0.77330	0.18548	-0.04952	-0.20784	0.19899	-0.07696
y	-0.14523	-0.40832	0.02859	0.29391	0.15281	0.19899
z	-0.32126	-0.42282	-0.67292	-0.08796	0.01508	-0.02612

e_{ij}^{elc} (C/m^2) using the LCALCEPS = .True. and k-point mesh of $3 \times 3 \times 3$:

	XX	YY	ZZ	XY	YZ	ZX
x	0.00094	-0.00053	0.00980	-0.14867	-0.01571	0.09045
y	-0.14794	0.14770	-0.00566	0.00073	0.07231	-0.01571
z	0.06682	0.05533	-0.24098	-0.00995	0.00585	-0.01013

C_{ij} (kBar) [1 kBar = 10^8 N/m^2] using IBRION = 6, k-point mesh of $6 \times 6 \times 6$:

Direction	XX	YY	ZZ	XY	YZ	ZX
XX	6057.3056	724.3785	180.7065	77.1802	-57.5022	-108.4995
YY	724.3785	6362.0550	168.1696	186.7406	113.4841	11.5359
ZZ	180.7065	168.1696	569.0700	-10.8572	-3.8761	6.7137
XY	77.1802	186.7406	-10.8572	2805.9056	-60.0177	-16.1907
YZ	-57.5022	113.4841	-3.8761	-60.0177	117.0219	-32.2759
ZX	-108.4995	11.5359	6.7137	-16.1907	-32.2759	154.2909

d_{ij} (pm/V):

	XX	YY	ZZ	XY	YZ	ZX
x	1.61207	-0.16238	-1.12554	-0.89758	17.67869	5.67306
y	0.15720	-0.99418	0.64978	1.77442	26.08633	17.67863
z	0.06403	-0.16243	-16.01638	-0.39226	0.84313	-1.46015

Convergence of e_{ij}^{ion}, e_{ij}^{elc}, C_{ij}, and d_{ij} in terms of k -point mesh (cutoff energy for the plane-wave-basis set, ENCUT = 500 eV):

e_{ij}^{ion} (C/m^2) using DFPT and k-point mesh of $3 \times 3 \times 3$:

	XX	YY	ZZ	XY	YZ	ZX
x	0.77562	0.15217	-0.06292	-0.19395	0.21631	-0.06870
y	-0.10759	-0.42770	0.03631	0.32188	0.18039	0.21763
z	-0.38117	-0.47927	-0.70580	-0.08496	0.01849	-0.03321

e_{ij}^{elc} (C/m^2) using DFPT and k-point mesh of $3 \times 3 \times 3$:

	XX	YY	ZZ	XY	YZ	ZX
x	0.00230	0.00362	0.01146	-0.14924	-0.01587	0.09071
y	-0.15171	0.14829	-0.00662	-0.00066	0.07239	-0.01587
z	0.06826	0.05644	-0.24302	-0.01023	0.00606	-0.01050

e_{ij}^{ion} (C/m^2) using DFPT and k-point mesh of $6 \times 6 \times 6$:

	XX	YY	ZZ	XY	YZ	ZX
x	0.77088	0.17608	-0.06625	-0.20151	0.20337	-0.07673
y	-0.13850	-0.40777	0.03822	0.30989	0.15692	0.20474
z	-0.37975	-0.47569	-0.70950	-0.08309	0.01848	-0.03236

e_{ij}^{elc} (C/m^2) using DFPT and k-point mesh of $6 \times 6 \times 6$:

	XX	YY	ZZ	XY	YZ	ZX
x	0.00297	0.00207	0.01163	-0.14887	-0.01572	0.09036
y	-0.14981	0.14690	-0.00671	0.00045	0.07222	-0.01572
z	0.06835	0.05654	-0.24231	-0.01023	0.00656	-0.01136

e_{ij}^{ion} (C/m^2) using DFPT and k-point mesh of $9 \times 9 \times 9$:

	XX	YY	ZZ	XY	YZ	ZX
x	0.77545	0.17622	-0.06663	-0.20340	0.20485	-0.07620
y	-0.13700	-0.41233	0.03846	0.30806	0.15977	0.20591
z	-0.37686	-0.47341	-0.71015	-0.08361	0.01818	-0.03247

e_{ij}^{elc} (C/m^2) using DFPT and k-point mesh of $9 \times 9 \times 9$:

	XX	YY	ZZ	XY	YZ	ZX
x	0.00296	0.00206	0.01163	-0.14887	-0.01572	0.09037
y	-0.14980	0.14690	-0.00672	0.00045	0.07222	-0.01572
z	0.06834	0.05653	-0.24231	-0.01023	0.00656	-0.01136

C_{ij} (kBar) [1 kBar = 10^8 N/m^2] using IBRION = 6 and k-point mesh of $3 \times 3 \times 3$:

Direction	XX	YY	ZZ	XY	YZ	ZX
XX	6012.3344	705.9739	181.2872	80.0248	-71.4231	-101.1577
YY	705.9739	6334.8203	169.6714	199.2562	115.8820	24.1527
ZZ	181.2872	169.6714	568.1644	-10.0596	-3.4187	5.9214
XY	80.0248	199.2562	-10.0596	2802.6400	-62.6552	-21.3045
YZ	-71.4231	115.8820	-3.4187	-62.6552	108.3261	-35.0541
ZX	-101.1577	24.1527	5.9214	-21.3045	-35.0541	148.8031

C_{ij} (kBar) [1 kBar = 10^8 N/m^2] using IBRION = 6 and k-point mesh of $6 \times 6 \times 6$:

Direction	XX	YY	ZZ	XY	YZ	ZX
XX	6057.3056	724.3785	180.7065	77.1802	-57.5022	-108.4995
YY	724.3785	6362.0550	168.1696	186.7406	113.4841	11.5359
ZZ	180.7065	168.1696	569.0700	-10.8572	-3.8761	6.7137
XY	77.1802	186.7406	-10.8572	2805.9056	-60.0177	-16.1907
YZ	-57.5022	113.4841	-3.8761	-60.0177	117.0219	-32.2759
ZX	-108.4995	11.5359	6.7137	-16.1907	-32.2759	154.2909

C_{ij} (kBar) [1 kBar = 10^8 N/m^2] using IBRION = 6 and k-point mesh of $9 \times 9 \times 9$:

Direction XX YY ZZ XY YZ ZX

XX 6027.2945 700.5691 193.1849 81.2582 -61.0995 -104.1367

YY 700.5691 6343.1964 179.2561 192.3208 112.4039 15.2749

ZZ 193.1849 179.2561 568.9964 -12.0628 -2.9642 5.1341

XY 81.2582 192.3208 -12.0628 2806.4602 -59.7058 -17.8094

YZ -61.0995 112.4039 -2.9642 -59.7058 115.2096 -32.0594

ZX -104.1367 15.2749 5.1341 -17.8094 -32.0594 152.2286

----

d_{ij} (pm/V) using e_{ij} (C/m^2) using DFPT and C_{ij} using IBRION = 6 and k-point mesh of $3 \times 3 \times 3$:

XX YY ZZ XY YZ ZX

---

x 1.77830 -0.32956 -1.33636 -0.70158 22.14008 7.90988

y 0.43763 -1.27434 0.77110 2.14348 33.44038 22.21708

z -0.00995 -0.22199 -16.60902 -0.37423 1.09557 -2.04273

---

d_{ij} (pm/V) using e_{ij} (C/m^2) using DFPT and C_{ij} using IBRION = 6 and k-point mesh of $6 \times 6 \times 6$:

XX YY ZZ XY YZ ZX

---

x 1.62693 -0.17683 -1.38574 -0.86611 18.11592 5.79975

y 0.17644 -1.013602 0.79938 1.84644 26.63895 18.18228

z -0.01048 -0.22295 -16.63361 -0.36970 1.08269 -1.91283

---

d_{ij} (pm/V) using e_{ij} (C/m^2) using DFPT and C_{ij} using IBRION = 6 and k-point mesh of $9 \times 9 \times 9$:

	XX	YY	ZZ	XY	YZ	ZX
x	1.66255	-0.18026	-1.44922	-0.86266	18.64831	5.96154
y	0.19606	-1.05225	0.83672	1.87233	27.46475	18.70833
z	0.02554	-0.19254	-16.67067	-0.38251	1.14157	-2.08452

Convergence in terms of the cutoff energy for the plane-wave-basis set (ENCUT):

e_{ij}^{ion} (C/m^2) using DFPT, k-point mesh of $6 \times 6 \times 6$, and ENCUT=500 eV:

	XX	YY	ZZ	XY	YZ	ZX
x	0.77088	0.17608	-0.06625	-0.20151	0.20337	-0.07673
y	-0.13850	-0.40777	0.03822	0.30989	0.15692	0.20474
z	-0.37975	-0.47569	-0.70950	-0.08309	0.01848	-0.03236

e_{ij}^{elc} (C/m^2) using DFPT, k-point mesh of $6 \times 6 \times 6$, and ENCUT=500 eV:

	XX	YY	ZZ	XY	YZ	ZX
x	0.00297	0.00207	0.01163	-0.14887	-0.01572	0.09036
y	-0.14981	0.14690	-0.00671	0.00045	0.07222	-0.01572
z	0.06835	0.05654	-0.24231	-0.01023	0.00656	-0.01136

C_{ij} (kBar) [1 kBar = 10^8 N/m^2] using IBRION = 6, k-point mesh of 6 × 6 × 6, and ENCUT=500 eV:

Direction	XX	YY	ZZ	XY	YZ	ZX
XX	6057.3056	724.3785	180.7065	77.1802	-57.5022	-108.4995
YY	724.3785	6362.0550	168.1696	186.7406	113.4841	11.5359
ZZ	180.7065	168.1696	569.0700	-10.8572	-3.8761	6.7137
XY	77.1802	186.7406	-10.8572	2805.9056	-60.0177	-16.1907
YZ	-57.5022	113.4841	-3.8761	-60.0177	117.0219	-32.2759
ZX	-108.4995	11.5359	6.7137	-16.1907	-32.2759	154.2909

d_{ij} (pm/V) from e_{ij} (C/m^2) using DFPT and C_{ij} using IBRION = 6, k-point mesh of 6 × 6 × 6, and ENCUT=500 eV:

	XX	YY	ZZ	XY	YZ	ZX
x	1.62693	-0.17683	-1.38574	-0.86611	18.11592	5.79975
y	0.17644	-1.013602	0.79938	1.84644	26.63895	18.18228
z	-0.01048	-0.22295	-16.63361	-0.36970	1.08269	-1.91283

e_{ij}^{ion} (C/m^2) using DFPT, k-point mesh of 6 × 6 × 6, and ENCUT=550 eV:

	XX	YY	ZZ	XY	YZ	ZX
x	0.76992	0.17663	-0.06513	-0.20278	0.20213	-0.07815
y	-0.14049	-0.40554	0.03758	0.30907	0.15406	0.20349
z	-0.38212	-0.47798	-0.70955	-0.08301	0.01875	-0.03283

e_{ij}^{elc} (C/m^2) using DFPT, k-point mesh of 6 × 6 × 6, and ENCUT=550 eV:

	XX	YY	ZZ	XY	YZ	ZX
x	0.00295	0.00207	0.01165	-0.14889	-0.01571	0.09040
y	-0.14982	0.14692	-0.00673	0.00044	0.07226	-0.01571
z	0.06835	0.05653	-0.24231	-0.01023	0.00656	-0.01136

C_{ij} (kBar) [1 kBar = 10^8 N/m^2] using IBRION = 6, k-point mesh of $6 \times 6 \times 6$, and ENCUT=550 eV:

Direction	XX	YY	ZZ	XY	YZ	ZX
XX	6013.1079	709.8334	161.4192	77.6200	-57.0112	-107.3486
YY	709.8334	6318.1008	149.0785	186.5116	112.3553	11.4899
ZZ	161.4192	149.0785	509.2360	-10.6874	-3.6157	6.2626
XY	77.6200	186.5116	-10.6874	2790.7541	-59.4192	-16.0718
YZ	-57.0112	112.3553	-3.6157	-59.4192	99.9454	-31.4769
ZX	-107.3486	11.4899	6.2626	-16.0718	-31.4769	136.2918

d_{ij} (pm/V) from e_{ij} (C/m^2) using DFPT and C_{ij} using IBRION = 6, k-point mesh of $6 \times 6 \times 6$, and ENCUT=550 eV:

	XX	YY	ZZ	XY	YZ	ZX
x	1.69866	-0.24815	-1.46786	-0.79374	21.65284	7.23228
y	0.295020	-1.13224	0.84654	1.98919	32.14055	21.72422
z	-0.017199	-0.23302	-18.59114	-0.37579	1.21262	-2.14621

e_{ij}^{ion} (C/m^2) using DFPT, k-point mesh of $6 \times 6 \times 6$, and ENCUT=600 eV:

	XX	YY	ZZ	XY	YZ	ZX
x	0.77169	0.17760	-0.06520	-0.20275	0.20210	-0.07813
y	-0.14082	-0.40680	0.03761	0.30951	0.15406	0.20347
z	-0.37997	-0.47601	-0.70993	-0.08318	0.01876	-0.03284

e_{ij}^{elc} (C/m^2) using DFPT, k-point mesh of $6 \times 6 \times 6$, and ENCUT=600 eV:

	XX	YY	ZZ	XY	YZ	ZX
x	0.00295	0.00207	0.01166	-0.14888	-0.01571	0.09041
y	-0.14982	0.14692	-0.00673	0.00044	0.07227	-0.01571
z	0.06836	0.05655	-0.24228	-0.01023	0.00656	-0.01137

C_{ij} (kBar) [1 kBar = 10^8 N/m^2] using IBRION = 6, k-point mesh of $6 \times 6 \times 6$, and ENCUT=600 eV:

Direction	XX	YY	ZZ	XY	YZ	ZX
XX	6014.5845	699.8289	164.1354	76.3296	-56.9357	-105.5981
YY	699.8289	6318.3877	151.5028	186.7717	110.5142	12.7974
ZZ	164.1354	151.5028	504.7936	-10.9401	-3.1740	5.4975
XY	76.3296	186.7717	-10.9401	2797.0924	-59.1977	-15.3693
YZ	-56.9357	110.5142	-3.1740	-59.1977	99.9006	-31.1389
ZX	-105.5981	12.7974	5.4975	-15.3693	-31.1389	135.8567

d_{ij} (pm/V) from e_{ij} (C/m^2) using DFPT and C_{ij} using IBRION = 6, k-point mesh of $6 \times 6 \times 6$, and ENCUT=600 eV:

	XX	YY	ZZ	XY	YZ	ZX
x	1.69680	-0.23634	-1.50092	-0.79700	21.59983	7.16638
y	0.28262	-1.12564	0.86577	1.97561	32.015359	21.67267
z	-0.00264	-0.21981	-18.77251	-0.37820	1.26064	-2.22971

Protruded $Ga_{0.0625}B_{0.9375}N$ Structure ($2 \times 2 \times 2$ supercell):

FE-$Ga_{0.0625}B_{0.9375}N$

1.00000000000000

5.0846566404208877 -0.0000000000000000 -0.0000000000000000

-2.5423283202104439 4.4034418201257228 0.0000000000000000

0.0000000000000000 0.0000000000000000 13.1696790696420383

B N Ga

15 16 1

Direct

x	y	z	Z_{33}	$\frac{du_3}{d\eta_3}$
0.1666938248494120	0.3333061751505879	0.9866885550600487	0.89311	-0.01766
0.6666123503011829	0.3333061751505879	0.9866885550600487	0.89311	-0.01766
0.1666938248494120	0.8333876496988171	0.9866885550600487	0.89311	-0.01766
0.6666666666666714	0.8333333333333286	0.9868387212922707	0.85848	-0.01856
0.1648003178679724	0.3351996821320274	0.4829178540592264	0.42648	0.04970
0.6703993642640690	0.3351996821320274	0.4829178540592264	0.42648	0.04970
0.1648003178679724	0.8296006357359310	0.4829178540592264	0.42648	0.04970
0.3333333333333286	0.1666666666666714	0.2386425776548698	0.81703	0.01750
0.8331272753066429	0.1662545506133069	0.2389143543644584	0.89170	0.01310
0.3337454493866933	0.6668727246933571	0.2389143543644584	0.89170	0.01310
0.8331272753066429	0.6668727246933571	0.2389143543644584	0.89170	0.01310
0.3333333333333286	0.1666666666666714	0.7350638005549202	0.98169	-0.03996
0.8354769358629500	0.1709538717259138	0.7344831777784866	0.94552	-0.04913
0.3290461282740862	0.6645230641370500	0.7344831777784866	0.94552	-0.04913
0.8354769358629500	0.6645230641370500	0.7344831777784866	0.94552	-0.04913
0.3333333333333286	0.1666666666666714	0.9856621203654377	-0.87262	-0.01287
0.8333196508617342	0.1666393017234828	0.9853587374572196	-0.88349	-0.00964
0.3333606982765174	0.6666803491382658	0.9853587374572196	-0.88349	-0.00964

0.8333196508617342	0.6666803491382658	0.9853587374572196	-0.88349	-0.00964
0.3333333333333286	0.1666666666666714	0.4905752760327685	-0.76662	0.03123
0.8463059168878423	0.1926118337756994	0.4820720591403263	-0.73974	0.05390
0.3073881662243006	0.6536940831121577	0.4820720591403263	-0.73974	0.05390
0.8463059168878423	0.6536940831121577	0.4820720591403263	-0.73974	0.05390
0.1666901479490377	0.3333098520509626	0.2377275483612485	-0.89113	0.02170
0.6666197041019394	0.3333098520509626	0.2377275483612485	-0.89113	0.02170
0.1666901479490377	0.8333802958980606	0.2377275483612485	-0.89113	0.02170
0.6666666666666714	0.8333333333333286	0.2369191466141073	-0.83759	0.02239
0.1665884367332164	0.3334115632667837	0.7363812378474074	-0.76470	-0.04699
0.6668231265335746	0.3334115632667837	0.7363812378474074	-0.76470	-0.04699
0.1665884367332164	0.8331768734664254	0.7363812378474074	-0.76470	-0.04699
0.6666666666666714	0.8333333333333286	0.7271602187711083	-1.47343	-0.03112
0.6666666666666714	0.8333333333333286	0.5455075665092600	1.67127	-0.01352

e_{ij}^{ion} (C/m^2) using the LCALCEPS = .True. and k-point mesh of $3 \times 3 \times 3$:

	XX	YY	ZZ	XY	YZ	ZX
x	-0.00000	-0.00000	0.00000	-0.13485	0.00000	-0.10090
y	-0.13485	0.13485	-0.00000	-0.00000	-0.10090	0.00000
z	-0.25820	-0.25820	-0.13544	0.00000	-0.00000	-0.00000

e_{ij}^{elc} (C/m^2) using the LCALCEPS = .True. and k-point mesh of $3 \times 3 \times 3$:

	XX	YY	ZZ	XY	YZ	ZX
x	0.00000	0.00000	0.00000	-0.04630	0.00000	0.06254
y	-0.04630	0.04630	-0.00000	0.00000	0.06254	0.00000
z	0.05606	0.05606	-0.15704	0.00000	-0.00000	0.00000

C_{ij} (kBar) [1 kBar = 10^8 N/m^2] using IBRION = 6, k-point mesh of 6 × 6 × 6:

Direction	XX	YY	ZZ	XY	YZ	ZX
XX	7618.1617	1505.1755	22.5941	-0.0000	-5.0934	-0.0000
YY	1505.1755	7618.1617	22.5941	0.0000	5.0934	-0.0000
ZZ	22.5941	22.5941	398.5747	0.0000	-0.0000	0.0000
XY	-0.0000	-0.0000	-0.0000	3056.4931	0.0000	-5.0934
YZ	-5.0934	5.0934	-0.0000	0.0000	79.4885	0.0000
ZX	0.0000	-0.0000	0.0000	-5.0934	0.0000	79.4885

d_{ij} (pm/V):

	XX	YY	ZZ	XY	YZ	ZX
x	0.00000	0.00000	0.00000	-0.60078	0.00000	-4.86435
y	-0.30039	0.30039	0.00000	0.00000	-4.86435	0.00000
z	-0.20345	-0.20345	-7.31508	0.00000	0.00000	0.00000

Convergence of e_{ij}^{ion}, e_{ij}^{elc}, C_{ij}, and d_{ij} in terms of k -point mesh (cutoff energy for the plane-wave-basis set, ENCUT = 500 eV):

e_{ij}^{ion} (C/m^2) using DFPT and k-point mesh of 3 × 3 × 3:

	XX	YY	ZZ	XY	YZ	ZX
x	0.00008	-0.00008	0.00000	-0.12789	0.00001	-0.09254
y	-0.12789	0.12790	-0.00000	0.00000	-0.09209	0.00000
z	-0.26388	-0.26388	-0.14281	-0.00000	0.00000	-0.00000

e_{ij}^{elc} (C/m^2) using DFPT and k-point mesh of $3 \times 3 \times 3$:

	XX	YY	ZZ	XY	YZ	ZX
x	0.00000	0.00000	0.00000	-0.05783	0.00000	0.05327
y	-0.05783	0.05783	-0.00000	0.00000	0.05327	0.00000
z	0.05736	0.05736	-0.15871	-0.00000	0.00000	0.00000

C_{ij} (kBar) [1 kBar = 10^8 N/m^2] using IBRION = 6 and k-point mesh of $3 \times 3 \times 3$:

Direction	XX	YY	ZZ	XY	YZ	ZX
XX	7610.4222	1504.1696	25.7035	0.0000	-6.5342	-0.0000
YY	1504.1696	7610.4222	25.7035	0.0000	6.5342	0.0000
ZZ	25.7035	25.7035	405.5043	-0.0000	0.0000	-0.0000
XY	-0.0000	0.0000	-0.0000	3053.1263	-0.0000	-6.5343
YZ	-6.5342	6.5342	0.0000	-0.0000	79.5009	-0.0000
ZX	-0.0000	0.0000	-0.0000	-6.5343	-0.0000	79.5008

d_{ij} (pm/V) using e_{ij} (C/m^2) using DFPT and C_{ij} using IBRION = 6 and k-point mesh of $3 \times 3 \times 3$:

	XX	YY	ZZ	XY	YZ	ZX
x	0.00013	-0.00013	0.00000	-0.61898	0.00128	-4.99045
y	-0.30943	0.30944	-0.00000	0.00000	-4.93383	0.00000
z	-0.20569	-0.20569	-7.40960	0.00000	0.00000	0.00000

e_{ij}^{ion} (C/m^2) using DFPT and k-point mesh of $6 \times 6 \times 6$:

	XX	YY	ZZ	XY	YZ	ZX
x	0.00005	-0.00005	-0.00000	-0.12949	0.00001	-0.09431
y	-0.12949	0.12950	0.00000	-0.00000	-0.09388	-0.00000
z	-0.26345	-0.26345	-0.14242	-0.00000	0.00000	-0.00000

e_{ij}^{elc} (C/m^2) using DFPT and k-point mesh of $6 \times 6 \times 6$:

	XX	YY	ZZ	XY	YZ	ZX
x	-0.00000	-0.00000	0.00000	-0.05013	0.00000	0.05913
y	-0.05013	0.05013	0.00000	0.00000	0.05913	0.00000
z	0.05394	0.05394	-0.15770	0.00000	-0.00000	0.00000

C_{ij} (kBar) [1 kBar = 10^8 N/m^2] using IBRION = 6 and k-point mesh of $6 \times 6 \times 6$:

Direction	XX	YY	ZZ	XY	YZ	ZX
XX	7618.1617	1505.1755	22.5941	-0.0000	-5.0934	-0.0000
YY	1505.1755	7618.1617	22.5941	0.0000	5.0934	-0.0000
ZZ	22.5941	22.5941	398.5747	0.0000	-0.0000	0.0000
XY	-0.0000	-0.0000	-0.0000	3056.4931	0.0000	-5.0934
YZ	-5.0934	5.0934	-0.0000	0.0000	79.4885	0.0000
ZX	0.0000	-0.0000	0.0000	-5.0934	0.0000	79.4885

d_{ij} (pm/V) using e_{ij} (C/m^2) using DFPT and C_{ij} using IBRION = 6 and k-point mesh of $6 \times 6 \times 6$:

	XX	YY	ZZ	XY	YZ	ZX
x	0.00008	-0.00008	0.00000	-0.59511	0.00127	-4.46393
y	-0.29751	0.29752	-0.00000	0.00000	-4.40983	0.00000
z	-0.21105	-0.21105	-7.50590	0.00000	0.00000	0.00000

e_{ij}^{ion} (C/m^2) using DFPT and k-point mesh of $9 \times 9 \times 9$:

	XX	YY	ZZ	XY	YZ	ZX
x	0.00002	-0.00002	-0.00000	-0.12837	0.00000	-0.09340
y	-0.12837	0.12837	0.00000	-0.00000	-0.09287	-0.00000
z	-0.26753	-0.26753	-0.14502	-0.00000	0.00000	-0.00000

e_{ij}^{elc} (C/m^2) using DFPT and k-point mesh of $9 \times 9 \times 9$:

	XX	YY	ZZ	XY	YZ	ZX
x	0.00000	0.00000	0.00000	-0.05013	0.00000	0.05923
y	-0.05013	0.05013	0.00000	0.00000	0.05923	0.00000
z	0.05384	0.05384	-0.15768	0.00000	0.00000	0.00000

C_{ij} (kBar) [1 kBar = 10^8 N/m^2] using IBRION = 6 and k-point mesh of $9 \times 9 \times 9$:

Direction	XX	YY	ZZ	XY	YZ	ZX
XX	7617.3106	1504.4460	21.7042	-0.0000	-5.2342	0.0000
YY	1504.4460	7617.3106	21.7042	-0.0000	5.2342	0.0000
ZZ	21.7042	21.7042	397.7057	-0.0000	0.0000	-0.0000
XY	-0.0000	-0.0000	-0.0000	3056.4323	0.0000	-5.2342
YZ	-5.2342	5.2342	0.0000	0.0000	79.4254	0.0000
ZX	-0.0000	0.0000	-0.0000	-5.2342	0.0000	79.4253

d_{ij} (pm/V) using e_{ij} (C/m^2) using DFPT and C_{ij} using IBRION = 6 and k-point mesh of $9 \times 9 \times 9$:

	XX	YY	ZZ	XY	YZ	ZX
x	0.00003	-0.00003	-0.00000	-0.59145	0.00000	-4.34113
y	-0.29567	0.29567	0.00000	0.00000	-4.27439	0.00000
z	-0.21621	-0.21621	-7.58756	0.00000	0.00000	0.00000

Convergence in terms of the cutoff energy for the plane-wave-basis set (ENCUT):

e_{ij}^{ion} (C/m^2) using DFPT, k-point mesh of $6 \times 6 \times 6$, and ENCUT=500 eV:

	XX	YY	ZZ	XY	YZ	ZX
x	0.00005	-0.00005	-0.00000	-0.12949	0.00001	-0.09431
y	-0.12949	0.12950	0.00000	-0.00000	-0.09388	-0.00000
z	-0.26345	-0.26345	-0.14242	-0.00000	0.00000	-0.00000

e_{ij}^{elc} (C/m^2) using DFPT, k-point mesh of $6 \times 6 \times 6$, and ENCUT=500 eV:

	XX	YY	ZZ	XY	YZ	ZX
x	-0.00000	-0.00000	0.00000	-0.05013	0.00000	0.05913
y	-0.05013	0.05013	0.00000	0.00000	0.05913	0.00000
z	0.05394	0.05394	-0.15770	0.00000	-0.00000	0.00000

C_{ij} (kBar) [1 kBar = 10^8 N/m^2] using IBRION = 6, k-point mesh of $6 \times 6 \times 6$, and ENCUT=500 eV:

Direction	XX	YY	ZZ	XY	YZ	ZX
XX	7618.1617	1505.1755	22.5941	-0.0000	-5.0934	-0.0000
YY	1505.1755	7618.1617	22.5941	0.0000	5.0934	-0.0000
ZZ	22.5941	22.5941	398.5747	0.0000	-0.0000	0.0000
XY	-0.0000	-0.0000	-0.0000	3056.4931	0.0000	-5.0934
YZ	-5.0934	5.0934	-0.0000	0.0000	79.4885	0.0000
ZX	0.0000	-0.0000	0.0000	-5.0934	0.0000	79.4885

d_{ij} (pm/V) from e_{ij} (C/m^2) using DFPT and C_{ij} using IBRION = 6, k-point mesh of $6 \times 6 \times 6$, and ENCUT=500 eV:

	XX	YY	ZZ	XY	YZ	ZX
x	0.00008	-0.00008	0.00000	-0.59511	0.00127	-4.46393
y	-0.29751	0.29752	-0.00000	0.00000	-4.40983	0.00000
z	-0.21105	-0.21105	-7.50590	0.00000	0.00000	0.00000

e_{ij}^{ion} (C/m^2) using DFPT, k-point mesh of $6 \times 6 \times 6$, and ENCUT=550 eV:

	XX	YY	ZZ	XY	YZ	ZX
x	0.00005	-0.00005	-0.00000	-0.12986	0.00001	-0.09442
y	-0.12986	0.12987	0.00000	0.00000	-0.09399	0.00000
z	-0.26385	-0.26385	-0.14273	-0.00000	-0.00000	-0.00000

e_{ij}^{elc} (C/m^2) using DFPT, k-point mesh of $6 \times 6 \times 6$, and ENCUT=550 eV:

	XX	YY	ZZ	XY	YZ	ZX
x	0.00000	0.00000	0.00000	-0.05014	0.00000	0.05918
y	-0.05014	0.05014	0.00000	0.00000	0.05918	0.00000
z	0.05392	0.05392	-0.15767	-0.00000	-0.00000	-0.00000

C_{ij} (kBar) [1 kBar = 10^8 N/m^2] using IBRION = 6, k-point mesh of $6 \times 6 \times 6$, and ENCUT=550 eV:

Direction	XX	YY	ZZ	XY	YZ	ZX
XX	7581.6480	1496.5085	8.7631	-0.0000	-5.7931	0.0000
YY	1496.5085	7581.6480	8.7631	0.0000	5.7931	-0.0000
ZZ	8.7631	8.7631	343.0201	-0.0000	-0.0000	-0.0000
XY	-0.0000	0.0000	0.0000	3042.5697	0.0000	-5.7931
YZ	-5.7931	5.7931	-0.0000	0.0000	61.5828	0.0000
ZX	0.0000	-0.0000	-0.0000	-5.7931	0.0000	61.5828

d_{ij} (pm/V) from e_{ij} (C/m^2) using DFPT and C_{ij} using IBRION = 6, k-point mesh of $6 \times 6 \times 6$, and ENCUT=550 eV:

	XX	YY	ZZ	XY	YZ	ZX
x	0.00008	-0.00008	-0.00000	-0.60261	0.00164	-5.77906
y	-0.30124	0.30125	-0.00000	0.00000	-5.70923	0.00000
z	-0.22280	-0.22280	-8.74612	0.00000	0.00000	0.00000

e_{ij}^{ion} (C/m^2) using DFPT, k-point mesh of $6 \times 6 \times 6$, and ENCUT=600 eV:

	XX	YY	ZZ	XY	YZ	ZX
x	0.00005	-0.00005	-0.00000	-0.13084	0.00001	-0.09537
y	-0.13083	0.13085	0.00000	0.00000	-0.09498	0.00000
z	-0.26405	-0.26405	-0.14298	-0.00000	-0.00000	-0.00000

e_{ij}^{elc} (C/m^2) using DFPT, k-point mesh of $6 \times 6 \times 6$, and ENCUT=600 eV:

	XX	YY	ZZ	XY	YZ	ZX
x	-0.00000	-0.00000	0.00000	-0.05014	0.00000	0.05918
y	-0.05014	0.05014	0.00000	0.00000	0.05918	0.00000
z	0.05393	0.05393	-0.15765	0.00000	0.00000	0.00000

C_{ij} (kBar) [1 kBar = 10^8 N/m^2] using IBRION = 6, k-point mesh of $6 \times 6 \times 6$, and ENCUT=600 eV:

Direction	XX	YY	ZZ	XY	YZ	ZX
XX	7592.2176	1500.3703	9.4854	-0.0000	-5.8092	-0.0000
YY	1500.3703	7592.2176	9.4854	-0.0000	5.8092	0.0000
ZZ	9.4854	9.4854	339.1457	-0.0000	0.0000	0.0000
XY	-0.0000	0.0000	0.0000	3045.9236	-0.0000	-5.8092
YZ	-5.8092	5.8092	-0.0000	0.0000	61.6225	-0.0000
ZX	0.0000	0.0000	0.0000	-5.8092	-0.0000	61.6225

d_{ij} (pm/V) from e_{ij} (C/m^2) using DFPT and C_{ij} using IBRION = 6, k-point mesh of $6 \times 6 \times 6$, and ENCUT=600 eV:

	XX	YY	ZZ	XY	YZ	ZX
x	0.00008	-0.00008	-0.00000	-0.60548	0.00164	-5.92993
y	-0.30267	0.30269	-0.00000	0.00000	-5.86663	0.00000
z	-0.22185	-0.22185	-8.85192	0.00000	0.00000	0.00000

Protruded $Ga_{0.1250}B_{0.8750}N$ Structure ($2 \times 2 \times 2$ supercell):

FE-$Ga_{0.1250}B_{0.8750}N$

1.00000000000000

5.1319845275475311 -0.0003644751788522 -0.0546951632669028

-2.5663079085377061 4.4442467350954225 0.0546951632669028

-0.1311999555185888 0.0757483296363242 12.6758813781508497

B N Ga

14 16 2

Direct

x	y	z	Z_{33}	$\frac{du_3}{d\eta_3}$
0.6713654039190177	0.3181609247297276	0.0023631259263193	0.55948	0.06691
0.1818390752702722	0.8286345960809823	0.0023631259263193	0.55948	0.06691
0.6720844612252124	0.8279155387747876	-0.0085213672712457	0.33152	0.04374
0.1720844612252117	0.3279155387747884	0.4914786327287543	0.33152	0.04374
0.6818390752702719	0.3286345960809824	0.5023631259263198	0.55948	0.06691
0.1713654039190176	0.8181609247297281	0.5023631259263198	0.55948	0.06691
0.3279572788686808	0.1720427211313195	0.2560436910399764	1.36973	-0.05646
0.8199788207474453	0.1720088733255804	0.2579744147004810	1.45026	-0.07790
0.3279911266744195	0.6800211792525547	0.2579744147004810	1.45026	-0.07790
0.8249626239926444	0.6750373760073556	0.2571424591216376	0.97998	-0.04096
0.3249626239926443	0.1750373760073555	0.7571424591216378	0.97998	-0.04096
0.8279911266744199	0.1800211792525546	0.7579744147004812	1.45026	-0.07790
0.3199788207474450	0.6720088733255801	0.7579744147004812	1.45026	-0.07790
0.8279572788686803	0.6720427211313197	0.7560436910399766	1.36973	-0.05646
0.3633580382848700	0.1366419617151303	0.0296137061838647	-1.14144	0.10944
0.8168958821214523	0.1472377878068432	-0.0171098538474203	-1.21321	0.05347
0.3527622121931569	0.6831041178785477	-0.0171098538474203	-1.21321	0.05347
0.8413944109779781	0.6586055890220219	0.0020647547046935	-0.99784	0.05120

0.3413944109779773	0.1586055890220223	0.5020647547046934	-0.99784	0.05120
0.8527622121931572	0.1831041178785466	0.4828901461525796	-1.21321	0.05347
0.3168958821214537	0.6472377878068428	0.4828901461525796	-1.21321	0.05347
0.8633580382848702	0.6366419617151298	0.5296137061838648	-1.14144	0.10944
0.1578370452989371	0.3421629547010629	0.2439364478952777	-1.92115	-0.02019
0.6585841876445486	0.3417858740298339	0.2583794776217299	-0.99948	-0.04137
0.1582141259701660	0.8414158123554514	0.2583794776217299	-0.99948	-0.04137
0.6584042316660947	0.8415957683339053	0.2553548938818408	-0.96590	-0.03662
0.1584042316660949	0.3415957683339051	0.7553548938818407	-0.96590	-0.03662
0.6582141259701660	0.3414158123554517	0.7583794776217293	-0.99948	-0.04137
0.1585841876445483	0.8417858740298340	0.7583794776217293	-0.99948	-0.04137
0.6578370452989368	0.8421629547010632	0.7439364478952771	-1.92115	-0.02019
0.1663710751451046	0.3336289248548956	0.0611510856417362	2.75393	-0.05234
0.6663710751451044	0.8336289248548956	0.5611510856417367	2.75393	-0.05234

e_{ij}^{ion} (C/m^2) using the LCALCEPS = .True. and k-point mesh of $3 \times 3 \times 3$:

	XX	YY	ZZ	XY	YZ	ZX
x	0.38273	-0.16919	-0.02023	-0.15688	0.17603	-0.16560
y	0.10013	-0.22342	0.01168	0.27596	0.03767	0.17603
z	-0.49124	-0.62104	-0.77589	-0.11241	0.03127	-0.05416

e_{ij}^{elc} (C/m^2) using the LCALCEPS = .True. and k-point mesh of $3 \times 3 \times 3$:

	XX	YY	ZZ	XY	YZ	ZX
x	-0.05269	-0.00642	0.06636	-0.07994	-0.02510	0.15235
y	-0.08959	0.12371	-0.03831	-0.02314	0.12337	-0.02510
z	0.12357	0.11899	-0.38816	-0.00397	0.00292	-0.00506

C_{ij} (kBar) [1 kBar = 10^8 N/m^2] using IBRION = 6, k-point mesh of $6 \times 6 \times 6$:

Direction	XX	YY	ZZ	XY	YZ	ZX
XX	5811.0393	798.8149	145.6305	-4.9146	-90.2868	-150.0868
YY	798.8149	6068.5033	147.6673	227.8849	157.2060	34.1794
ZZ	145.6305	147.6673	478.3188	1.7639	-2.3815	4.1250
XY	-4.9146	227.8849	1.7639	2704.8851	-92.1331	-17.3603
YZ	-90.2868	157.2060	-2.3815	-92.1331	124.5254	-28.8723
ZX	-150.0868	34.1794	4.1250	-17.3603	-28.8723	157.8643

d_{ij} (pm/V):

	XX	YY	ZZ	XY	YZ	ZX
x	0.94882	-0.81220	0.97440	-0.30183	14.27614	2.79095
y	0.82640	-0.90531	-0.56250	1.76104	19.27668	14.27637
z	-0.04030	-0.24592	-24.21409	-0.35617	1.64116	-2.84269

Convergence of e_{ij}^{ion}, e_{ij}^{elc}, C_{ij}, and d_{ij} in terms of k -point mesh (cutoff energy for the plane-wave-basis set, ENCUT = 500 eV):

e_{ij}^{ion} (C/m^2) using DFPT and k-point mesh of $3 \times 3 \times 3$:

	XX	YY	ZZ	XY	YZ	ZX
x	0.36755	-0.17102	-0.01546	-0.15990	0.17377	-0.16384
y	0.09192	-0.20503	0.00892	0.27354	0.03500	0.17524
z	-0.51483	-0.63038	-0.80014	-0.10007	0.03352	-0.06217

e_{ij}^{elc} (C/m^2) using DFPT and k-point mesh of $3 \times 3 \times 3$:

	XX	YY	ZZ	XY	YZ	ZX
x	-0.05742	-0.00071	0.06479	-0.07964	-0.02597	0.15320
y	-0.09560	0.12916	-0.03741	-0.02835	0.12321	-0.02597
z	0.12479	0.11944	-0.39147	-0.00463	0.00323	-0.00560

C_{ij} (kBar) [1 kBar = 10^8 N/m^2] using IBRION = 6 and k-point mesh of $3 \times 3 \times 3$:

Direction	XX	YY	ZZ	XY	YZ	ZX
XX	5819.6079	823.4044	141.1392	-7.1183	-98.0362	-151.7545
YY	823.4044	6077.0713	141.8932	230.0882	163.1683	38.9424
ZZ	141.1392	141.8932	476.1720	0.6530	-2.3976	4.1529
XY	-7.1183	230.0882	0.6530	2699.4189	-95.3484	-20.5033
YZ	-98.0362	163.1683	-2.3976	-95.3484	118.6514	-32.2518
ZX	-151.7545	38.9424	4.1529	-20.5033	-32.2518	155.8925

d_{ij} (pm/V) using e_{ij} (C/m^2) using DFPT and C_{ij} using IBRION = 6 and k-point mesh of $3 \times 3 \times 3$:

	XX	YY	ZZ	XY	YZ	ZX
x	0.97955	-0.86290	1.04865	-0.24564	15.24994	3.58132
y	0.90103	-0.96602	-0.60760	1.85276	21.04437	15.30724
z	-0.09083	-0.25867	-24.88216	-0.32624	1.68834	-3.40180

e_{ij}^{ion} (C/m^2) using DFPT and k-point mesh of $6 \times 6 \times 6$:

	XX	YY	ZZ	XY	YZ	ZX
x	0.37658	-0.16790	-0.01781	-0.15862	0.17166	-0.16566
y	0.09206	-0.21222	0.01027	0.27833	0.03063	0.17415
z	-0.50533	-0.61917	-0.80406	-0.09859	0.03376	-0.06056

e_{ij}^{elc} (C/m^2) using DFPT and k-point mesh of $6 \times 6 \times 6$:

	XX	YY	ZZ	XY	YZ	ZX
x	-0.05485	-0.00618	0.06577	-0.08129	-0.02514	0.15277
y	-0.09177	0.12700	-0.03797	-0.02433	0.12374	-0.02514
z	0.12488	0.12059	-0.39212	-0.00372	0.00353	-0.00611

C_{ij} (kBar) [1 kBar $= 10^8$ N/m^2] using IBRION $= 6$ and k-point mesh of $6 \times 6 \times 6$:

Direction	XX	YY	ZZ	XY	YZ	ZX
XX	5811.0393	798.8149	145.6305	-4.9146	-90.2868	-150.0868
YY	798.8149	6068.5033	147.6673	227.8849	157.2060	34.1794
ZZ	145.6305	147.6673	478.3188	1.7639	-2.3815	4.1250
XY	-4.9146	227.8849	1.7639	2704.8851	-92.1331	-17.3603
YZ	-90.2868	157.2060	-2.3815	-92.1331	124.5254	-28.8723
ZX	-150.0868	34.1794	4.1250	-17.3603	-28.8723	157.8643

d_{ij} (pm/V) using e_{ij} (C/m^2) using DFPT and C_{ij} using IBRION $= 6$ and k-point mesh of $6 \times 6 \times 6$:

	XX	YY	ZZ	XY	YZ	ZX
x	0.92187	-0.79417	1.01401	-0.33035	13.83826	2.69998
y	0.78306	-0.85360	-0.58848	1.73434	18.55249	13.96764
z	-0.05791	-0.22447	-24.88277	-0.30394	1.77269	-3.28873

e_{ij}^{ion} (C/m^2) using DFPT and k-point mesh of $9 \times 9 \times 9$:

	XX	YY	ZZ	XY	YZ	ZX
x	0.37853	-0.16886	-0.01787	-0.15987	0.17318	-0.16552
y	0.09351	-0.21447	0.01032	0.27742	0.03287	0.17446
z	-0.50354	-0.61905	-0.80441	-0.10004	0.03357	-0.06159

e_{ij}^{elc} (C/m^2) using DFPT and k-point mesh of $9 \times 9 \times 9$:

	XX	YY	ZZ	XY	YZ	ZX
x	-0.05484	-0.00621	0.06577	-0.08126	-0.02514	0.15278
y	-0.09172	0.12697	-0.03797	-0.02432	0.12375	-0.02514
z	0.12488	0.12060	-0.39214	-0.00370	0.00353	-0.00611

C_{ij} (kBar) [1 kBar = 10^8 N/m^2] using IBRION = 6 and k-point mesh of $9 \times 9 \times 9$:

Direction	XX	YY	ZZ	XY	YZ	ZX
XX	5812.5270	799.5556	144.7495	-5.7400	-90.1505	-150.3775
YY	799.5556	6068.5631	146.8509	227.4737	157.3050	34.0626
ZZ	144.7495	146.8509	477.7061	1.8198	-2.4303	4.2094
XY	-5.7400	227.4737	1.8198	2705.1407	-92.2201	-17.2412
YZ	-90.1505	157.3050	-2.4303	-92.2201	124.5508	-28.8959
ZX	-150.3775	34.0626	4.2094	-17.2412	-28.8959	157.9168

d_{ij} (pm/V) using e_{ij} (C/m^2) using DFPT and C_{ij} using IBRION = 6 and k-point mesh of $9 \times 9 \times 9$:

	XX	YY	ZZ	XY	YZ	ZX
x	0.92943	-0.80112	1.01554	-0.32848	13.98436	2.74705
y	0.79282	-0.86511	-0.58791	1.73998	18.77499	14.03831
z	-0.06014	-0.22468	-24.92106	-0.31048	1.72563	-3.34973

Convergence in terms of the cutoff energy for the plane-wave-basis set (ENCUT):

e_{ij}^{ion} (C/m^2) using DFPT, k-point mesh of $6 \times 6 \times 6$, and ENCUT=500 eV:

	XX	YY	ZZ	XY	YZ	ZX
x	0.37658	-0.16790	-0.01781	-0.15862	0.17166	-0.16566
y	0.09206	-0.21222	0.01027	0.27833	0.03063	0.17415
z	-0.50533	-0.61917	-0.80406	-0.09859	0.03376	-0.06056

e_{ij}^{elc} (C/m^2) using DFPT, k-point mesh of $6 \times 6 \times 6$, and ENCUT=500 eV:

	XX	YY	ZZ	XY	YZ	ZX
x	-0.05485	-0.00618	0.06577	-0.08129	-0.02514	0.15277
y	-0.09177	0.12700	-0.03797	-0.02433	0.12374	-0.02514
z	0.12488	0.12059	-0.39212	-0.00372	0.00353	-0.00611

C_{ij} (kBar) [1 kBar = 10^8 N/m^2] using IBRION = 6, k-point mesh of $6 \times 6 \times 6$, and ENCUT=500 eV:

Direction	XX	YY	ZZ	XY	YZ	ZX
XX	5811.0393	798.8149	145.6305	-4.9146	-90.2868	-150.0868
YY	798.8149	6068.5033	147.6673	227.8849	157.2060	34.1794
ZZ	145.6305	147.6673	478.3188	1.7639	-2.3815	4.1250
XY	-4.9146	227.8849	1.7639	2704.8851	-92.1331	-17.3603
YZ	-90.2868	157.2060	-2.3815	-92.1331	124.5254	-28.8723
ZX	-150.0868	34.1794	4.1250	-17.3603	-28.8723	157.8643

d_{ij} (pm/V) from e_{ij} (C/m^2) using DFPT and C_{ij} using IBRION = 6, k-point mesh of $6 \times 6 \times 6$, and ENCUT=500 eV:

	XX	YY	ZZ	XY	YZ	ZX
x	0.92187	-0.79417	1.01401	-0.33035	13.83826	2.69998
y	0.78306	-0.85360	-0.58848	1.73434	18.55249	13.96764
z	-0.05791	-0.22447	-24.88277	-0.30394	1.77269	-3.28873

e_{ij}^{ion} (C/m^2) using DFPT, k-point mesh of $6 \times 6 \times 6$, and ENCUT=550 eV:

	XX	YY	ZZ	XY	YZ	ZX
x	0.37699	-0.16775	-0.01779	-0.15919	0.17119	-0.16618
y	0.09148	-0.21197	0.01027	0.27846	0.02956	0.17367
z	-0.50621	-0.62019	-0.80445	-0.09871	0.03367	-0.06041

e_{ij}^{elc} (C/m^2) using DFPT, k-point mesh of $6 \times 6 \times 6$, and ENCUT=550 eV:

	XX	YY	ZZ	XY	YZ	ZX
x	-0.05490	-0.00619	0.06580	-0.08128	-0.02513	0.15284
y	-0.09176	0.12703	-0.03799	-0.02435	0.12382	-0.02513
z	0.12488	0.12057	-0.39216	-0.00373	0.00353	-0.00612

C_{ij} (kBar) [1 kBar = 10^8 N/m^2] using IBRION = 6, k-point mesh of $6 \times 6 \times 6$, and ENCUT=550 eV:

Direction	XX	YY	ZZ	XY	YZ	ZX
XX	5764.0782	782.9547	126.4482	-4.4134	-90.2719	-149.2853
YY	782.9547	6022.3427	127.9075	228.0771	156.7113	34.2089
ZZ	126.4482	127.9075	419.2293	1.2638	-2.4494	4.2425
XY	-4.4134	228.0771	1.2638	2689.3564	-91.7471	-17.5512
YZ	-90.2719	156.7113	-2.4494	-91.7471	107.3374	-28.2702
ZX	-149.2853	34.2089	4.2425	-17.5512	-28.2702	139.9810

d_{ij} (pm/V) from e_{ij} (C/m^2) using DFPT and C_{ij} using IBRION = 6, k-point mesh of $6 \times 6 \times 6$, and ENCUT=550 eV:

	XX	YY	ZZ	XY	YZ	ZX
x	1.00560	-0.88633	1.17304	-0.23049	16.52772	3.60950
y	0.93687	-1.00212	-0.68075	1.90971	22.54762	16.66922
z	-0.06927	-0.23505	-28.40239	-0.30609	1.90257	-3.56255

e_{ij}^{ion} (C/m^2) using DFPT, k-point mesh of $6 \times 6 \times 6$, and ENCUT=600 eV:

	XX	YY	ZZ	XY	YZ	ZX
x	0.37717	-0.16798	-0.01785	-0.15915	0.17110	-0.16611
y	0.09177	-0.21222	0.01030	0.27868	0.02952	0.17358
z	-0.50573	-0.61981	-0.80449	-0.09879	0.03373	-0.06051

e_{ij}^{elc} (C/m^2) using DFPT, k-point mesh of $6 \times 6 \times 6$, and ENCUT=600 eV:

	XX	YY	ZZ	XY	YZ	ZX
x	-0.05491	-0.00619	0.06580	-0.08126	-0.02513	0.15284
y	-0.09175	0.12703	-0.03799	-0.02436	0.12382	-0.02513
z	0.12490	0.12060	-0.39214	-0.00373	0.00353	-0.00612

C_{ij} (kBar) [1 kBar = 10^8 N/m^2] using IBRION = 6, k-point mesh of $6 \times 6 \times 6$, and ENCUT=600 eV:

Direction	XX	YY	ZZ	XY	YZ	ZX
XX	5774.5701	784.6248	128.3421	-5.5544	-89.9296	-149.3887
YY	784.6248	6031.4548	129.2737	228.0230	156.4421	34.1857
ZZ	128.3421	129.2737	415.8037	0.8068	-2.3085	3.9985
XY	-5.5544	228.0230	0.8068	2694.0498	-91.7872	-17.1991
YZ	-89.9296	156.4421	-2.3085	-91.7872	107.7529	-28.1390
ZX	-149.3887	34.1857	3.9985	-17.1991	-28.1390	140.2450

d_{ij} (pm/V) from e_{ij} (C/m^2) using DFPT and C_{ij} using IBRION = 6, k-point mesh of $6 \times 6 \times 6$, and ENCUT=600 eV:

	XX	YY	ZZ	XY	YZ	ZX
x	0.99932	-0.88115	1.17601	-0.23400	16.41683	3.56473
y	0.92881	-0.993405	-0.68247	1.89822	22.37455	16.55809
z	-0.05567	-0.22418	-28.64604	-0.31059	1.92071	-3.59162
